# Burden of laryngeal cancer in China caused by smoking from 1990 to 2021 and predictions for 2035: An age-period-cohort analysis of global burden of disease study 2021

**DOI:** 10.18332/tid/202875

**Published:** 2025-04-14

**Authors:** Xue Gu, Xiaopeng Sun, Xiao Ren, Yu Li, Yingying Fang, Hui Song, Pingli Luo, Mengfan Yuan

**Affiliations:** 1 Department of Otolaryngology Head and Neck Surgery, Second Affiliated Hospital of Xi'an Medical University, Xi 'an, China

**Keywords:** smoking, laryngeal cancer, disability-adjusted life-years, APC, BAPC

## Abstract

**INTRODUCTION:**

Smoking is a major risk factor for laryngeal cancer (LC). Understanding the impact of smoking on the changing disease burden of LC is crucial for LC prevention in China and provides a scientific basis for formulating targeted LC prevention and control strategies, contributing to the achievement of the ‘Healthy China 2030’ goals.

**METHODS:**

Data on LC attributable to smoking in China, stratified by sex, age, and year, were obtained from the 2021 Global Burden of Disease (GBD) study to conduct a secondary data analysis. Joinpoint regression was used to analyze trends in the burden of LC attributable to smoking in China from 1990 to 2021. Age-period-cohort (APC) analysis was employed to compare and analyze trends in the age, period, and cohort effects on the disease burden. Finally, Bayesian age-period-cohort (BAPC) analysis was used to predict trends in LC mortality and disability-adjusted life years (DALYs) from 2022 to 2035.

**RESULTS:**

From 1990 to 2021, the overall burden of LC attributable to smoking in China increased. The number of deaths in males rose from 9128 to 14219, and in females from 790 to 1054. DALYs increased by 39.85% in males and 22.21% in females. Despite the rise in absolute burden, age-standardized mortality rates (ASMR) and age-standardized DALY rates (ASDR) declined, with reductions exceeding 50% in females. Joinpoint regression analysis revealed a decline-stabilization-decline trend in age-standardized rates among males, while females exhibited a continuous decline. According to the APC model, the age effect on disease burden increased with age, while period and cohort risk ratios generally declined. Net drift analysis showed a decline in mortality and DALY rates attributable to smoking, more pronounced in females than males, with local drift values <0 for both sexes. Predictions indicate that by 2035, male LC deaths will reach 17205, and female deaths 1373; however, ASMR and ASDR will continue to decline, with male ASMR dropping to 2.44 per 100000 and female ASMR to 0.16 per 100000.

**CONCLUSIONS:**

Over the past three decades, the burden of LC attributable to smoking in China has shown an increasing trend, with sex and age disparities. This burden is expected to continue rising over the next fourteen years. Therefore, it is imperative to strengthen smoking prevention and cessation efforts, particularly targeting high-risk groups. Additionally, continued emphasis on education and awareness regarding LC is necessary to facilitate early detection and intervention, thereby effectively reducing the disease burden attributable to smoking.

## INTRODUCTION

About 30% of all new diagnoses in the head and neck cancer category are for laryngeal cancer (LC), making it the second most frequent type of cancer^1^. With 188960 new cases and 103216 fatalities worldwide in 2022^2^, it contributed significantly to the global health burden. Although the disease burden of LC in China remains relatively low, it still warrants significant attention. According to the latest cancer epidemiology data released in 2024, an estimated 29500 new cases of LC were diagnosed in China in 2022, with 16900 associated deaths^3^. Moreover, the majority of patients are already in the advanced stage at the time of initial diagnosis^4^, further underscoring the need for vigilance and effective intervention.

There are several risk factors for LC, and the majority of them may be controllable. Smoking is one of the most important and controllable risk factors for LC^5^. Smoking is responsible for about 70% of LC fatalities^1^. With >300 million regular smokers and a smoking rate of 26.6% among those aged ≥15 years^6^, China is the world’s biggest producer and user of tobacco products, accounting for >44% of worldwide tobacco consumption^7^. In recent years, smoking prevalence in China has shown a declining trend^8^. However, due to the country’s large population base, the number of smoking-related cases and deaths continues to rise. By 2019, China had become the country with the second-highest absolute number of smoking-attributable LC deaths and corresponding disability-adjusted life years (DALYs)^9^.

Based on the most recent data on China from the 2021 Global Burden of Disease (GBD) study, this study examines the epidemiological features of smoking-attributable LC in light of the poor survival rates of LC patients and the difficulty of LC prevention and control linked to smoking^10^. It analyzes its epidemiological features by estimating the general trends and parameters of smoking-induced LC death rates and DALYs in China from 1990 to 2021 using an age-period-cohort (APC) analytic model. To enhance decision-making for the prevention and control of LC in China, it also forecasts trends in smoking-attributable LC fatalities and DALYs from 2022 to 2035.

## METHODS

### Study design and data sources

The data for this study were retrieved from the GBD 2021 study. This study presents a secondary analysis of the dataset on the burden of LC attributable to smoking in China from 1990 to 2021, as provided by the GBD study. The GBD study utilized multiple data sources, including vital statistics, national health surveys, disease registries, and other health-related datasets. The GBD study employed the DisMod-MR 2.1 Bayesian meta-regression tool to comprehensively estimate disease incidence, prevalence, mortality, years of life lost (YLL), years lived with disability (YLD), and DALYs. These estimates were reported by time, location, age group, and sex^11^. Specifically, we downloaded the following data for subsequent analysis: the number of deaths and DALYs attributable to smoking-related LC in China from 1990 to 2021, stratified by sex and encompassing all age groups, as well as the corresponding age-standardized rates (ASRs), namely the age-standardized mortality rate (ASMR) and the age-standardized DALYs rate (ASDR). DALYs represent the total years of healthy life lost (YLL) due to premature death and disability (YLD), calculated as the sum of YLL and YLD (DALYs = YLL + YLD), quantifying the overall health impact of the disease. Data collection and access for this study occurred on 26 August 2024. Since publicly available databases were used, this study did not require ethical approval or informed consent.

### Definitions

In the GBD database, LC is classified under the ICD-10 code C32 (Malignant neoplasm of larynx), which includes the following subcategories: C32.0 (Glottis), C32.1 (Supraglottis), C32.3 (Laryngeal cartilage), C32.8 (Overlapping lesion of larynx), and C32.9 (Larynx, unspecified). The corresponding ICD-9 codes used are 161–161.9 and V10.21^11^. In the GBD study, smoking is categorized into active smoking and passive smoking (secondhand smoke). This study focuses on active smoking, defined as current use of any tobacco product or former smokers who quit at least six months prior^12^.

### Statistical analysis

*Descriptive study*

The number of deaths and DALYs across all age categories, together with the related age-standardized mortality rate (ASMR) and age-standardized DALYs rate (ASDR), were all included in this study’s descriptive analysis of the burden of smoking-attributable LC in China.

*Joinpoint regression analysis*

The Joinpoint regression model was employed to evaluate trends in disease burden over time. This model utilizes the least squares method to estimate the patterns of change in disease rates, thereby avoiding the non-objectivity inherent in typical trend analyses based on linear assumptions. By calculating the sum of squared residuals between the estimated and actual values, the model identifies the number of joinpoints where significant changes in mortality trends occur^13^. The Joinpoint software (version 4.9.1.0; National Cancer Institute, Rockville, Maryland, USA) was used to construct this model. Additionally, we calculated the annual percentage change and the average annual percentage change to investigate the fluctuation trends across different segments. The statistical significance of these trends was assessed by comparing the average annual percentage change to zero, with a significance level set at p<0.05.

*Age-period-cohort analysis (APC)*

Using an APC model framework, this study examined possible changes in smoking-attributable LC death and DALY rates by age, period, and birth cohort. We separated the data into 14 continuous age groups in the APC model, each lasting five years and ranging from 30–34 to ≥95 years (median: 65–69 years). Periods were separated into 6 groups every 5 years, from 1992–1996 to 2017–2021 (median: 2002–2006); similarly, birth cohorts were separated into 21 groups every 5 years, from 1895–1899 to 1995–1999 (median: 1940–1944). Age groups younger than 30 years are not accessible in the GBD database.

The longitudinal age curve, which shows the age-specific ratios in relation to the reference cohort after adjusting for period bias, is one of the outputs of the APC model that illustrates how age affects the trajectory of smoking-attributable LC. Indicating the influence of period and cohort effects on the trajectory of smoking-attributable LC, the rate ratios (RR) for period and cohort show the relative risks based on age-specific rates for the chosen reference period and cohort. The overall temporal trend of death and DALY rates is represented by net drift, which shows the annual percentage change taking period and cohort into account (%/year). The yearly percentage shift in death and DALY rates within certain age groups is known as local drift. A drift below 0.0% each year denotes a decline in percentage change for the subsequent year, but a drift over 0.0% per year is seen as a growth trend. For all analyses, R software (version 4.3.0) was used. Statistical significance was established at a threshold of p<0.05^14,15^.

*Bayesian age-period-cohort (BAPC) analysis*

Using the integrated nested Laplacian approximations (INLA, version 23.09.09) and BAPC (version 0.0.36) packages in R, a BAPC analysis was performed to estimate the number of deaths and DALYs attributable to smoking-induced LC in Chinese male and female populations between 2022 and 2035, as well as the ASMR and ASDR. The BAPC model employs a second-order random walk to smooth the priors for age, period, and cohort effects, enabling the prediction of posterior mortality rates^16^. The model utilizes INLA to approximate the marginal posterior distributions, thereby avoiding the mixing and convergence issues associated with Markov chain Monte Carlo sampling methods. In this study, an inverse-gamma prior was applied to the precision parameters, with the shape parameter and scale parameter both set to 0.001. Sensitivity analysis was conducted, and the predicted trends remained consistent under different priors, indicating robust model performance. Based on age-specific population data from 1990 to 2021, projected population data for 2022–2035, and the GBD’s world standard population age structure, the BAPC model was applied to predict the sex-specific trends and changes in the burden of LC attributable to smoking in China over the next 14 years.

## RESULTS

### Trends in smoking-attributable LC burden

Table 1 summarizes the overall trends in smoking-attributable LC burden in China from 1990 to 2021, including the number of deaths, DALYs, ASMR, and ASDR among both sexes. Males consistently had a greater burden of smoking-related illnesses than females, both in terms of quantity and age-standardized rates. Between 1990 and 2021, smoking-induced LC caused more deaths and DALYs for both sexes in China. For males, the number of deaths grew from 9128 cases to 14219 cases, a 55.77% rise, while for females, the increase was 33.42%. The burden of DALYs rose from 256508 years to 358738 years for males, a 39.85% increase, and from 18672 years to 22819 years for females, a 22.21% increase. Nonetheless, throughout these three decades, ASMR and ASDR declined in both sexes, with the percentage decline in females surpassing 50% (Table 1).

**Table 1:** Changes in smoking-attributable male and female deaths and DALYs, along with age-standardized rates (per 100000), for laryngeal cancer in China in 1990 and 2021

*Measure*	*Male*			*Female*		
	*1990 n (95% UI)*	*2021 n (95% UI)*	*Change %*	*1990 n (95% UI)*	*2021 n (95% UI)*	*Change %*
**Deaths**	9128 (7292–10936)	14219 (10545–18691)	55.78	790 (442–1071)	1054 (581–1551)	33.42
**DALYs**	256508 (202997–308036)	358738 (260942–474093)	39.85	18672 (10000–25419)	22819 (12810–33449)	22.21
**ASMR**	2.43 (1.97–2.90)	1.44 (1.08–1.87)	-40.76	0.20 (0.12–0.27)	0.09 (0.05–0.14)	-52.77
**ASDR**	58.87 (47.14–70.53)	33.79 (24.76–44.48)	-42.61	4.37 (2.41–5.93)	2.01 (1.13–2.96)	-53.91

DALYs: disability adjusted life years. UI: uncertainty interval. ASMR: age-standardized mortality rate. ASDR: age-standardized DALYs rate.

**Figure 1:**
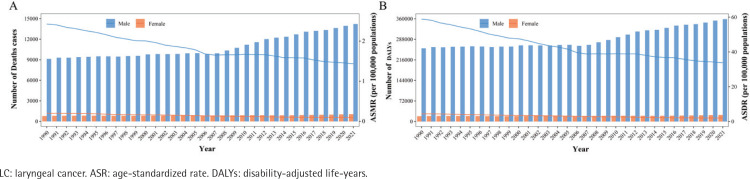
The trends in the number of deaths and DALYs attributable to smoking-induced LC in China from 1990 to 2021, along with the corresponding ASR per 100000, show the changing patterns among males (blue) and females (orange). The bar graph illustrates the number, while the line graph represents the ASR: A) Deaths; and B) DALYs

To investigate the yearly changes in the number of deaths and DALYs linked to smoking-induced LC, as well as ASMR and ASDR among both sexes in China from 1990 to 2021, we next grouped the data by year (Figure 1, and Supplementary file Table 1). Between 1990 and 2021, the number of deaths for Chinese males from smoking-induced LC increased overall, peaking at 14219 instances in 2021 (Figure 1A). Comparably, DALYs showed a similar rising pattern, with minor oscillations from 1990 to 2006 before a consistent rise that peaked in 2021 at 358738 years (Figure 1B). Similar to the pattern in DALYs for males, the number of deaths in females attributable to smoking-induced LC varied somewhat between 1990 and 2006 before steadily rising to a peak of 1054 cases in 2021 (Figure 1A). Between 1990 and 2014, the DALY trend varied significantly, but it thereafter gradually increased, peaking at 22819 years in 2021 (Figure 1B).

In terms of variations in ASRs, the ASMR for Chinese males first decreased, then stabilized, and then declined even more. From 2.43 per 100000 in 1990 to 1.65 per 100000 in 2007, the ASMR steadily decreased to 1.44 per 100000 in 2021, after stabilizing at 1.66 per 100000 from 2008 to 2012 (Figure 1A). From 1990 to 2006, the ASDR fell; from 2007 to 2013, it fluctuated little; and in 2021, it continuously dropped to 33.79 per 100000 (Figure 1B). Both the ASMR and ASDR load changes for females demonstrated a steady and ongoing decrease (Figures 1A and 1B).

### Joinpoint regression analysis of the disease burden of LC attributable to smoking

**Figure 2:**
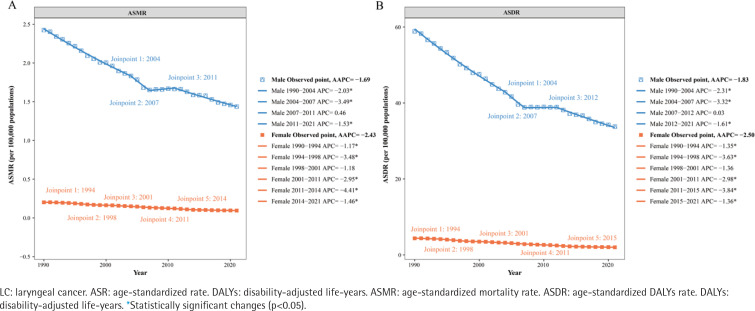
Joinpoint regression analysis of smoking-attributable LC ASMR and ASDR in China from 1990 to 2021: A) ASMR; and B) ASDR. The analysis includes data for males (blue) and females (orange)

The Joinpoint regression analysis of the ASMR and ASDR for LC attributable to smoking in China from 1990 to 2021 revealed significant gender disparities (Figure 2, and Supplementary file Table 2). For males, the analysis identified three joinpoints, indicating a trend characterized by an initial decline, followed by a stabilization period, and then a subsequent continued decline. Specifically, from 1990 to 2007, both ASMR and ASDR in males exhibited a consistent downward trend. This was followed by a stabilization phase (with a slight increase from 2007 to 2012) and then a significant decline from 2013 to 2021 (Figures 2A and 2B). In contrast, the analysis for females identified five joinpoints, dividing the trend into six segments, with ASMR and ASDR showing a continuous decline throughout the entire period from 1990 to 2021 (Figures 2A and 2B).

### The impact of APC on smoking-attributable LC death rates and DALYs rates

**Figure 3:**
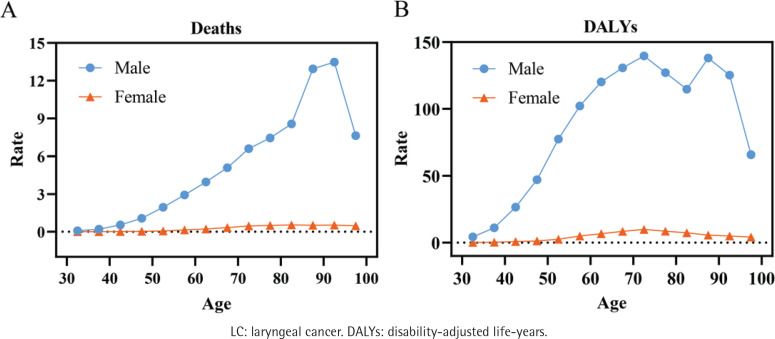
Estimated impact of age on smoking-attributable LC death rates and DALYs rates (/100000 personyears) in China: A) Deaths; and B) DALYs. The analysis includes data for males (blue) and females (orange)

Figure 3 and Supplementary file Tables 3–5 present the findings of the APC analysis on smoking-attributable LC death rates and DALYs rates in Chinese males and females.

In terms of age effects, death rates increase with age and peak in the 90–94 years age range. This trend is the same for both males and females (Figure 3A). Age-related increases in male DALY rates show an M-shaped pattern, peaking at 139.68 per 100000 in the age group of 70–74 years and then falling to 138.10 per 100000 in the age group of 85–89 years (Figure 3B). In contrast, this ratio rises with age for females, reaching a high of 9.89 per 100000 in the age range of 70–74 years (Figure 3B).

**Figure 4:**
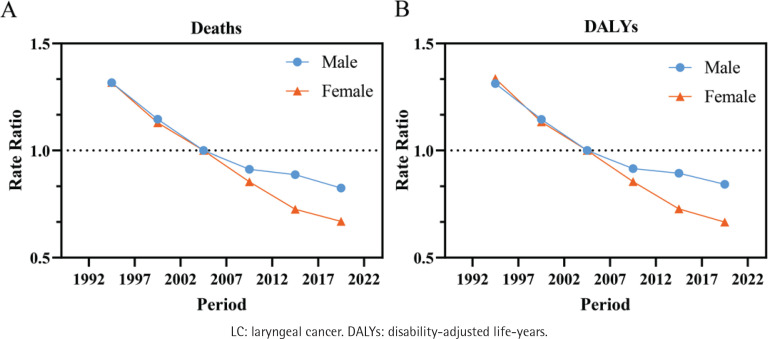
Estimated impact of periods on smoking-attributable LC death rates and DALYs rates (/100000 person-years) in China: A) Deaths; and B) DALYs. The analysis includes data for males (blue) and females (orange)

Male and female death risks have steadily declined during the research period, with the largest period effects in 1992–1996 (male: RR=1.32; 95% CI: 1.27–1.36; female: RR=1.32; 95% CI: 1.18–1.47) and the lowest in 2017–2021 (male: RR=0.83; 95% CI: 0.79–0.86; female: RR=0.67; 95% CI: 0.59–0.76) (Figure 4A). Comparable to death rates, DALYs also show a downward trend, dropping by 35.81% for males and 50.10% for females between 1992–1996 and 2017–2021 (Figure 4B).

In terms of cohort effects, for both Chinese males and females, the risk of smoking-attributable LC death rate declined as the number of birth cohorts increased throughout the 1895–1989 observation period. Males born in the 1910–1914 cohort had greater illness risks than those born in subsequent cohorts (RR=1.51; 95% CI: 1.40–1.63) (Supplementary file Figure 1A). The 1900–1904 cohort had the highest risk for females (RR=2.81; 95% CI: 1.50–5.28), whereas the 1975–1989 group had a steady risk (RR=0.36; 95% CI: 0.17–0.74) (Supplementary file Figure 1A ). Both sexes had a general declining trend of RR in the terms of DALYs, with males showing minor variations in the 1900–1904 and 1910–1914 cohorts (Supplementary file Figure 1B) and females steadily declining, with the 1985–1989 cohort showing the lowest risk value (RR=0.25; 95% CI: 0.14–0.44) (Supplementary file Figure 1B).

### Net drift and local drift values of smoking-attributable LC death rates and DALYs rates

The net drift and local drift values of smoking-attributable LC death rates and DALYs rates in China, stratified by sex, are shown in Supplementary file Figure 2 and Supplementary file Table 6. In China, both male and female death and DALY rates had negative net drift values; female deaths net drift value was -2.74% (95% CI: -3.34 – -2.14), which was substantially lower than that of the male (-1.81%; 95% CI: -2.02 – -1.60) (Supplementary file Figure 2A). This suggested that Chinese females had a larger overall drop in smoking-attributable LC death rates than Chinese males. The net drift value for females (-2.79%; 95% CI: -3.02 – -2.57) was much lower than that of males (-1.73%; 95% CI: -2.08 – -1.37) for DALYs rates, as seen in Supplementary file Figure 2B. Furthermore, the smoking-attributable LC death rates and DALYs rates in China over the last 30 years for both males and females in all age groups had local drift values <0, indicating a drop in these rates in China across all age groups (Supplementary file Figure 2).

### Prediction of smoking-attributable LC death and DALYs burden

We also employed BAPC to forecast the future trends of ASMR and ASDR as well as the death and DALY numbers of smoking-attributable LC in China in both males and females between 2022 and 2035 (Supplementary file Figure 3 and Supplementary file Table 7). Over the following 14 years, there is a steady upward trend in the number of deaths for both males and females, reaching a high in 2035 with 17205 cases for males and 1373 cases for females (Supplementary file Figure 3A). Both male and female ASMRs show a consistent downward trend. Male ASMR is predicted to reach 2.44/100000 (95% CI: 1.28–3.59) by 2035, whereas female ASMR is predicted to decline more gradually, reaching 0.16/100000 by 2035 (95% CI: 0.08–0.23) (Supplementary file Figure 3A).

Between 2022 and 2035, the number of DALYs increase significantly for both males and females, but the growth was more noticeable for females (female, 23.81% vs male, 4.97%). ASDRs for both sexes steadily decline, which is consistent with ASMR trends (Supplementary file Figure 3B).

## DISCUSSION

The examination of the shifting patterns in the burden of smoking-related LC in China from 1990 to 2021, based on the estimation findings of GBD 2021, reveals that ASDR and ASMR of LC have declined during the last three decades. However, the number of deaths and DALYs related to smoking-induced LC has grown due to the aging and growing Chinese population. Males account for the bulk of cases, and the DALY and death rates are 11–16 times higher for men than for women. According to the APC model, the risk of smoking-induced LC rises with age, with the lowest RR of death and DALYs between 2017 and 2021, and those born after 1945 have reduced death and DALYs risks. Additionally, the study’s forecasts reveal that, between 2022 and 2035, smoking-induced LC will be responsible for more deaths and DALYs, whereas ASDR and ASMR will decrease. This discrepancy suggests that aging and population expansion may eventually take center stage in the causes of smoking-induced LC-related deaths and DALYs.

The trends in smoking-attributable LC death rates and DALYs in China are shown in this study. Between 1990 and 2021, there was a notable decline in the ASDR and ASMR of smoking-induced LC, a development that would not have been feasible without state assistance. The World Health Organization Framework Convention on Tobacco Control (WHO FCTC) was signed by China in 2003, ratified in 2005, and went into effect in 2006^17^. The FCTC’s approval and implementation gave proponents of tobacco control a moral and legal advantage. China subsequently raised tobacco taxes twice between 2006 and 2015^18,19^; the first tax hike was implemented in May 2009, although it had no direct impact on cigarette prices at retail. Moreover, tobacco commercials were outlawed in 2015 by the Advertisement Law of the People’s Republic of China in outdoor areas, public transportation, and the media^20^. The marketing of tobacco goods online was prohibited under the 2016 Measures for the Management of Internet Advertising^21^. By 2022, 30% of the population should have complete smoke-free legal protection, and by 2030, more than 80% of the population should have complete smoke-free legal protection, according to the Healthy China Initiative (2019–2030)^22^. The promotion of smoke-free laws in nearby Chinese cities is another admirable move. Many Chinese provincial capitals, including large cities like Beijing and Shanghai with populations in the tens of millions, enacted smoke-free laws between 2007 and 2018^23,24^. The intentions and the existing state of affairs, however, differ greatly. Due to the State Tobacco Monopoly Administration’s strong interference and propensity to put business concerns ahead of social ones, comprehensive tobacco control measures in China have been developed and implemented slowly. Furthermore, as of 2018, the smoking prevalence among those aged ≥15 years was 26.6%^25^, a slight drop from 28.1% in 2010^26^, owing to China’s enormous population and aging demographics. As a result, China’s tobacco control initiatives continue to encounter formidable obstacles.

From 1990 to 2021, there were significant sex differences in the smoking-attributable disease burden of LC in China. Joinpoint regression analysis effectively illustrated the nonlinear trend of disease burden over time, revealing that the burden was substantially higher in males than in females, with distinct developmental patterns. These findings align with a previous global study on LC trends^27^. This is partially because men smoke at higher rates than women. According to research on smoking rates among Chinese people, men smoke up to 20 times more frequently than women^28,29^. Additionally, the impact of sex hormones may be the reason why LC is less common in women than in men. Sex-related estrogen receptors have been implicated in the formation and occurrence of LC, and estrogen receptor beta has been demonstrated to protect LC cells by controlling indicators of the epithelialmesenchymal transition, including nuclear betacatenin activation and E-cadherin downregulation^30,31^. In laryngeal squamous cell carcinoma, the lack of estrogen receptors is linked to a more invasive tumor^32^.

The APC model deconstructed and quantified the smoking-attributable LC burden in China across age, period, and birth cohort dimensions, providing a more comprehensive understanding of its disease burden trends. The age effect indicated that the risk of smoking-induced LC mortality and DALYs rates increased with age and over time. An obvious rise in adverse effects and disease risks can be attributed to aging populations, deteriorating physical performance and immunity, and the long-term cumulative consequences of smoking. Changes in particular medical technology, finances, and cultural norms that are peculiar to a given period are referred to as period effects. Between 2007 and 2011, China’s smoking-induced LC period RRs generally exhibited a downward trend in both DALYs and death rates. More healthcare resources, greater socioeconomic levels, and increased public health awareness may be the main causes of this notable decline. As previously stated, China has been enforcing certain anti-smoking laws since 2006, which set the stage for a decrease in period RRs.

This study found that the cohort effect exhibited an overall declining trend, with lower mortality risks observed in later birth cohorts, reflecting the evolving pattern of disease burden in China. This trend may be attributed to improvements in healthcare, the widespread adoption of early screening, the strengthening of tobacco control policies, and socioeconomic development^33,34^. From a historical perspective on disease patterns, infectious diseases (e.g. tuberculosis, typhoid) were the primary causes of death in the early 1900s. However, with the advent of vaccination programs and public health improvements, mortality rates from infectious diseases declined significantly. In the latter half of the 20th century, industrialization and lifestyle changes led to a shift, with chronic diseases (e.g. cardiovascular diseases, cancer) becoming the predominant causes of mortality. Notably, after the 1950s, a surge in tobacco consumption contributed to a growing burden of smoking-related diseases, such as lung cancer and LC. Within the birth cohorts analyzed in this study (1895–1985), earlier cohorts (1895–1945) exhibited a higher risk of LC. This may be linked to the lack of tobacco control measures, longer cumulative smoking exposure, and the adverse health impacts of a high infectious disease burden during youth. In contrast, later cohorts (1965–1985) benefited from stricter tobacco control policies, improved health education, and better access to medical resources, leading to a decline in smoking prevalence and a reduced disease burden. The APC analysis in this study elucidates the long-term impacts of age, period, and cohort effects on the burden of smoking-related LC. These findings underscore the critical importance of sustained tobacco control efforts, the promotion of early screening, and the optimization of prevention and treatment strategies to mitigate the burden of smoking-related cancers.

### Limitations

Our study has several limitations. First, although the GBD database has been widely applied and incorporates Bayesian models and multiple data integration methods to enhance data accuracy, discrepancies still exist between GBD estimates and national statistics. Second, since the GBD modeling approach primarily relies on regional and national-level projections, it does not provide specific data for different regions within China, limiting inter-regional comparisons and preventing the differentiation between urban and rural disparities. Third, this study assumes that the impact of smoking on LC is independent. However, due to data constraints, we were unable to distinguish between different types of smoking (e.g. cigarettes, cigars), which may affect risk assessment across smoking patterns. Fourth, the APC model is a descriptive analysis tool that, while effective in identifying temporal trends, cannot establish causal relationships. This limitation introduces the potential for ecological fallacy, restricting the applicability of the findings at the individual level. Finally, the estimates and projections in the GBD database rely on existing data and statistical assumptions, which may introduce residual confounding factors. Therefore, the results should be interpreted with caution.

## CONCLUSIONS

Between 1990 and 2021, China’s smoking-attributable LC disease burden remained high, with age and sex disparities, with males suffering from a much higher illness burden than females, and a disproportionately high burden among the elderly. Over the following 14 years, it is anticipated that while the corresponding ASR will decrease, the number of deaths and DALYs will continue to increase. Efforts should be increased to regulate and stop smoking, continuously enforce smoke-free rules in public areas, improve public education on LC, and raise resident knowledge and quality of screenings to enable early identification of precancerous lesions and early-stage cancer for prompt treatment. This also has important implications for reducing the burden of LC disease in the elderly population.

## Supplementary Material



## Data Availability

The data used in this study are available from the authors on reasonable request.
