# Supplementary material for: Burden of laryngeal cancer in China caused by smoking from 1990 to 2021 and predictions for 2035: An age-period-cohort analysis of global burden of disease study 2021
Source: Tob Induc Dis. 2025 Apr 14;23:10.18332/tid/202875. doi: 10.18332/tid/202875 (PMC11992923; doi:10.18332/tid/202875)
Supplement: Supplementary file 1 [file TID-23-47-s1.pdf]

Supplementary file Figure 1. Estimated impact of birth cohorts on smoking-attributable LC death rates and DALYs rates (/100,000 person-years) in China. (A) Deaths; (B) DALYs. The analysis includes data for males (blue line) and females (orange line). LC, laryngeal cancer; DALYs: disability-adjusted life-years.

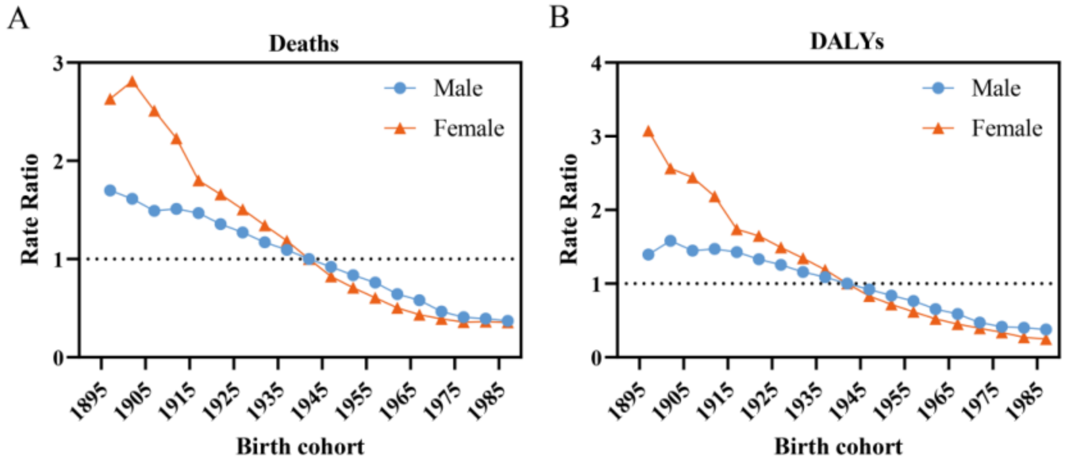

Supplementary file Figure 2. Net drift and local drift values of smoking-attributable LC death rates and DALYs rates (/100,000 person-years) in China from 1990 to 2021. (A) Deaths; (B) DALYs. The analysis includes data for males (blue line) and females (orange line). LC, laryngeal cancer; DALYs, disability-adjusted life-years.

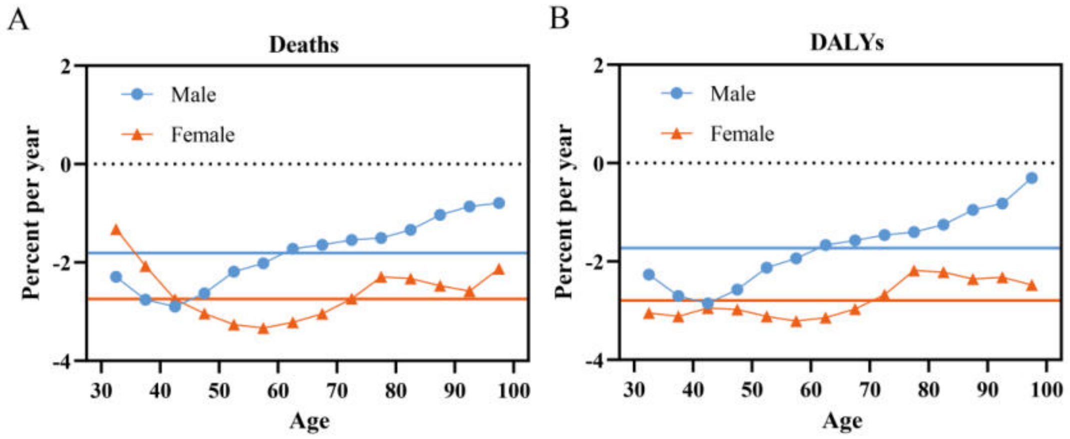

Supplementary file Figure 3. Time trends of smoking-attributable LC deaths and DALYs numbers in males and females in China from 2022 to 2035, along with the corresponding ASR/100,000. (A) Deaths; (B) DALYs. The analysis includes data for males (blue) and females (orange). The left Y-axis represents the count, shown as bar charts, while the right Y-axis represents ASR, shown as line segments. LC, laryngeal cancer; ASMR: Age-standardized mortality rate; ASDR: Age-standardized DALYs rate; DALYs: Disability-adjusted life-years

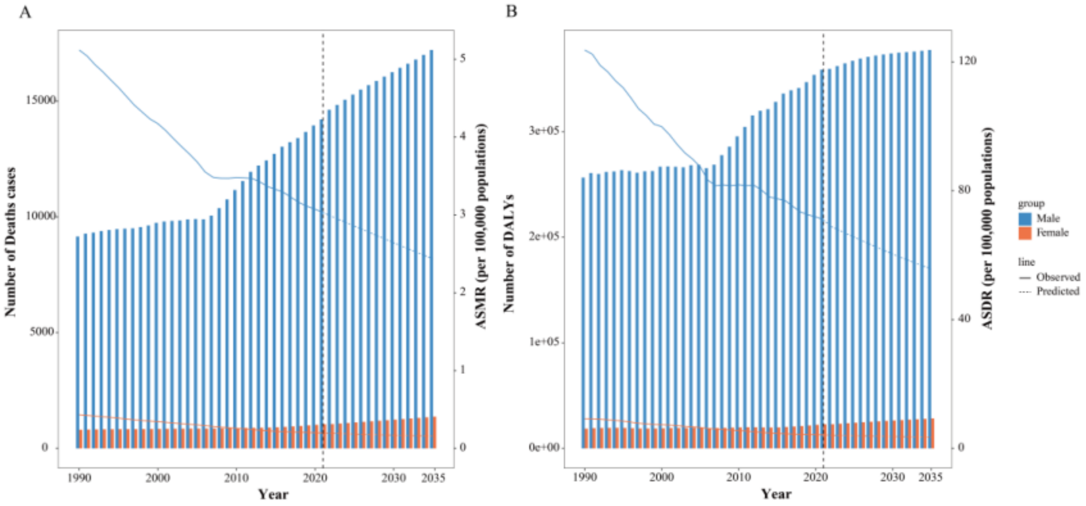

Supplementary Table 1: Number of deaths and disability-adjusted life years (DALYs) due to laryngeal cancer attributable to smoking by sex, and corresponding age-standardized rates (ASRs per 100,000) in China, 1990-2021.

measure	location	sex	age	cause	rei	metric	year	val
Deaths	China	Male	All ages	Larynx can	Smoking	Number	1990	9128
Deaths	China	Female	All ages	Larynx can	Smoking	Number	1990	790
Deaths	China	Male	All ages	Larynx can	Smoking	Number	1991	9306
Deaths	China	Female	All ages	Larynx can	Smoking	Number	1991	816
Deaths	China	Male	All ages	Larynx can	Smoking	Number	1992	9301
Deaths	China	Female	All ages	Larynx can	Smoking	Number	1992	829
Deaths	China	Male	All ages	Larynx can	Smoking	Number	1993	9394
Deaths	China	Female	All ages	Larynx can	Smoking	Number	1993	834
Deaths	China	Male	All ages	Larynx can	Smoking	Number	1994	9431
Deaths	China	Female	All ages	Larynx can	Smoking	Number	1994	840
Deaths	China	Male	All ages	Larynx can	Smoking	Number	1995	9497
Deaths	China	Female	All ages	Larynx can	Smoking	Number	1995	840
Deaths	China	Male	All ages	Larynx can	Smoking	Number	1996	9494
Deaths	China	Female	All ages	Larynx can	Smoking	Number	1996	831
Deaths	China	Male	All ages	Larynx can	Smoking	Number	1997	9465
Deaths	China	Female	All ages	Larynx can	Smoking	Number	1997	820
Deaths	China	Male	All ages	Larynx can	Smoking	Number	1998	9538
Deaths	China	Female	All ages	Larynx can	Smoking	Number	1998	823
Deaths	China	Male	All ages	Larynx can	Smoking	Number	1999	9580
Deaths	China	Female	All ages	Larynx can	Smoking	Number	1999	826
Deaths	China	Male	All ages	Larynx can	Smoking	Number	2000	9786
Deaths	China	Female	All ages	Larynx can	Smoking	Number	2000	839
Deaths	China	Male	All ages	Larynx can	Smoking	Number	2001	9832
Deaths	China	Female	All ages	Larynx can	Smoking	Number	2001	857
Deaths	China	Male	All ages	Larynx can	Smoking	Number	2002	9821
Deaths	China	Female	All ages	Larynx can	Smoking	Number	2002	857
Deaths	China	Male	All ages	Larynx can	Smoking	Number	2003	9849
Deaths	China	Female	All ages	Larynx can	Smoking	Number	2003	858
Deaths	China	Male	All ages	Larynx can	Smoking	Number	2004	9944
Deaths	China	Female	All ages	Larynx can	Smoking	Number	2004	870
Deaths	China	Male	All ages	Larynx can	Smoking	Number	2005	9967
Deaths	China	Female	All ages	Larynx can	Smoking	Number	2005	870
Deaths	China	Male	All ages	Larynx can	Smoking	Number	2006	9792
Deaths	China	Female	All ages	Larynx can	Smoking	Number	2006	850
Deaths	China	Male	All ages	Larynx can	Smoking	Number	2007	9944
Deaths	China	Female	All ages	Larynx can	Smoking	Number	2007	852
Deaths	China	Male	All ages	Larynx can	Smoking	Number	2008	10354
Deaths	China	Female	All ages	Larynx can	Smoking	Number	2008	864
Deaths	China	Male	All ages	Larynx can	Smoking	Number	2009	10747
Deaths	China	Female	All ages	Larynx can	Smoking	Number	2009	877
Deaths	China	Male	All ages	Larynx can	Smoking	Number	2010	11192
Deaths	China	Female	All ages	Larynx can	Smoking	Number	2010	889
Deaths	China	Male	All ages	Larynx can	Smoking	Number	2011	11574
Deaths	China	Female	All ages	Larynx can	Smoking	Number	2011	906
Deaths	China	Male	All ages	Larynx can	Smoking	Number	2012	12018
Deaths	China	Female	All ages	Larynx can	Smoking	Number	2012	900

Deaths	China	Male	All ages	Larynx can	Smoking	Number	2013	12230
Deaths	China	Female	All ages	Larynx can	Smoking	Number	2013	887
Deaths	China	Male	All ages	Larynx can	Smoking	Number	2014	12358
Deaths	China	Female	All ages	Larynx can	Smoking	Number	2014	882
Deaths	China	Male	All ages	Larynx can	Smoking	Number	2015	12710
Deaths	China	Female	All ages	Larynx can	Smoking	Number	2015	895
Deaths	China	Male	All ages	Larynx can	Smoking	Number	2016	13098
Deaths	China	Female	All ages	Larynx can	Smoking	Number	2016	926
Deaths	China	Male	All ages	Larynx can	Smoking	Number	2017	13221
Deaths	China	Female	All ages	Larynx can	Smoking	Number	2017	943
Deaths	China	Male	All ages	Larynx can	Smoking	Number	2018	13350
Deaths	China	Female	All ages	Larynx can	Smoking	Number	2018	967
Deaths	China	Male	All ages	Larynx can	Smoking	Number	2019	13637
Deaths	China	Female	All ages	Larynx can	Smoking	Number	2019	998
Deaths	China	Male	All ages	Larynx can	Smoking	Number	2020	13956
Deaths	China	Female	All ages	Larynx can	Smoking	Number	2020	1031
Deaths	China	Male	All ages	Larynx can	Smoking	Number	2021	14219
Deaths	China	Female	All ages	Larynx can	Smoking	Number	2021	1054
DALYs (D	China	Male	All ages	Larynx can	Smoking	Number	1990	256508
DALYs (D	China	Female	All ages	Larynx can	Smoking	Number	1990	18672
DALYs (D	China	Male	All ages	Larynx can	Smoking	Number	1991	260852
DALYs (D	China	Female	All ages	Larynx can	Smoking	Number	1991	19221
DALYs (D	China	Male	All ages	Larynx can	Smoking	Number	1992	259964
DALYs (D	China	Female	All ages	Larynx can	Smoking	Number	1992	19442
DALYs (D	China	Male	All ages	Larynx can	Smoking	Number	1993	261895
DALYs (D	China	Female	All ages	Larynx can	Smoking	Number	1993	19512
DALYs (D	China	Male	All ages	Larynx can	Smoking	Number	1994	262315
DALYs (D	China	Female	All ages	Larynx can	Smoking	Number	1994	19602
DALYs (D	China	Male	All ages	Larynx can	Smoking	Number	1995	263642
DALYs (D	China	Female	All ages	Larynx can	Smoking	Number	1995	19479
DALYs (D	China	Male	All ages	Larynx can	Smoking	Number	1996	262756
DALYs (D	China	Female	All ages	Larynx can	Smoking	Number	1996	19191
DALYs (D	China	Male	All ages	Larynx can	Smoking	Number	1997	261070
DALYs (D	China	Female	All ages	Larynx can	Smoking	Number	1997	18867
DALYs (D	China	Male	All ages	Larynx can	Smoking	Number	1998	262428
DALYs (D	China	Female	All ages	Larynx can	Smoking	Number	1998	18878
DALYs (D	China	Male	All ages	Larynx can	Smoking	Number	1999	262636
DALYs (D	China	Female	All ages	Larynx can	Smoking	Number	1999	18865
DALYs (D	China	Male	All ages	Larynx can	Smoking	Number	2000	266878
DALYs (D	China	Female	All ages	Larynx can	Smoking	Number	2000	19078
DALYs (D	China	Male	All ages	Larynx can	Smoking	Number	2001	267118
DALYs (D	China	Female	All ages	Larynx can	Smoking	Number	2001	19415
DALYs (D	China	Male	All ages	Larynx can	Smoking	Number	2002	266735
DALYs (D	China	Female	All ages	Larynx can	Smoking	Number	2002	19427
DALYs (D	China	Male	All ages	Larynx can	Smoking	Number	2003	266315
DALYs (D	China	Female	All ages	Larynx can	Smoking	Number	2003	19440
DALYs (D	China	Male	All ages	Larynx can	Smoking	Number	2004	268351
DALYs (D	China	Female	All ages	Larynx can	Smoking	Number	2004	19713
DALYs (D	China	Male	All ages	Larynx can	Smoking	Number	2005	268755
DALYs (D	China	Female	All ages	Larynx can	Smoking	Number	2005	19646

DALYs (D	China	Male	All ages	Larynx can	Smoking	Number	2006	265298
DALYs (D	China	Female	All ages	Larynx can	Smoking	Number	2006	19262
DALYs (D	China	Male	All ages	Larynx can	Smoking	Number	2007	268604
DALYs (D	China	Female	All ages	Larynx can	Smoking	Number	2007	19325
DALYs (D	China	Male	All ages	Larynx can	Smoking	Number	2008	277760
DALYs (D	China	Female	All ages	Larynx can	Smoking	Number	2008	19526
DALYs (D	China	Male	All ages	Larynx can	Smoking	Number	2009	285829
DALYs (D	China	Female	All ages	Larynx can	Smoking	Number	2009	19761
DALYs (D	China	Male	All ages	Larynx can	Smoking	Number	2010	295624
DALYs (D	China	Female	All ages	Larynx can	Smoking	Number	2010	19954
DALYs (D	China	Male	All ages	Larynx can	Smoking	Number	2011	304572
DALYs (D	China	Female	All ages	Larynx can	Smoking	Number	2011	20197
DALYs (D	China	Male	All ages	Larynx can	Smoking	Number	2012	315443
DALYs (D	China	Female	All ages	Larynx can	Smoking	Number	2012	20098
DALYs (D	China	Male	All ages	Larynx can	Smoking	Number	2013	319744
DALYs (D	China	Female	All ages	Larynx can	Smoking	Number	2013	19880
DALYs (D	China	Male	All ages	Larynx can	Smoking	Number	2014	321264
DALYs (D	China	Female	All ages	Larynx can	Smoking	Number	2014	19751
DALYs (D	China	Male	All ages	Larynx can	Smoking	Number	2015	328214
DALYs (D	China	Female	All ages	Larynx can	Smoking	Number	2015	19886
DALYs (D	China	Male	All ages	Larynx can	Smoking	Number	2016	336160
DALYs (D	China	Female	All ages	Larynx can	Smoking	Number	2016	20380
DALYs (D	China	Male	All ages	Larynx can	Smoking	Number	2017	339198
DALYs (D	China	Female	All ages	Larynx can	Smoking	Number	2017	20754
DALYs (D	China	Male	All ages	Larynx can	Smoking	Number	2018	341347
DALYs (D	China	Female	All ages	Larynx can	Smoking	Number	2018	21225
DALYs (D	China	Male	All ages	Larynx can	Smoking	Number	2019	346902
DALYs (D	China	Female	All ages	Larynx can	Smoking	Number	2019	21744
DALYs (D	China	Male	All ages	Larynx can	Smoking	Number	2020	353832
DALYs (D	China	Female	All ages	Larynx can	Smoking	Number	2020	22402
DALYs (D	China	Male	All ages	Larynx can	Smoking	Number	2021	358738
DALYs (D	China	Female	All ages	Larynx can	Smoking	Number	2021	22819
Deaths	China	Male	Age-standa	Larynx can	Smoking	Rate	1990	2,43
Deaths	China	Female	Age-standa	Larynx can	Smoking	Rate	1990	0,20
Deaths	China	Male	Age-standa	Larynx can	Smoking	Rate	1991	2,40
Deaths	China	Female	Age-standa	Larynx can	Smoking	Rate	1991	0,20
Deaths	China	Male	Age-standa	Larynx can	Smoking	Rate	1992	2,34
Deaths	China	Female	Age-standa	Larynx can	Smoking	Rate	1992	0,20
Deaths	China	Male	Age-standa	Larynx can	Smoking	Rate	1993	2,31
Deaths	China	Female	Age-standa	Larynx can	Smoking	Rate	1993	0,20
Deaths	China	Male	Age-standa	Larynx can	Smoking	Rate	1994	2,26
Deaths	China	Female	Age-standa	Larynx can	Smoking	Rate	1994	0,19
Deaths	China	Male	Age-standa	Larynx can	Smoking	Rate	1995	2,22
Deaths	China	Female	Age-standa	Larynx can	Smoking	Rate	1995	0,19
Deaths	China	Male	Age-standa	Larynx can	Smoking	Rate	1996	2,16
Deaths	China	Female	Age-standa	Larynx can	Smoking	Rate	1996	0,18
Deaths	China	Male	Age-standa	Larynx can	Smoking	Rate	1997	2,09
Deaths	China	Female	Age-standa	Larynx can	Smoking	Rate	1997	0,17
Deaths	China	Male	Age-standa	Larynx can	Smoking	Rate	1998	2,05
Deaths	China	Female	Age-standa	Larynx can	Smoking	Rate	1998	0,17

Deaths	China	Male	Age-standa	Larynx can	Smoking	Rate	1999	2,01
Deaths	China	Female	Age-standa	Larynx can	Smoking	Rate	1999	0,17
Deaths	China	Male	Age-standa	Larynx can	Smoking	Rate	2000	2,00
Deaths	China	Female	Age-standa	Larynx can	Smoking	Rate	2000	0,16
Deaths	China	Male	Age-standa	Larynx can	Smoking	Rate	2001	1,96
Deaths	China	Female	Age-standa	Larynx can	Smoking	Rate	2001	0,16
Deaths	China	Male	Age-standa	Larynx can	Smoking	Rate	2002	1,90
Deaths	China	Female	Age-standa	Larynx can	Smoking	Rate	2002	0,16
Deaths	China	Male	Age-standa	Larynx can	Smoking	Rate	2003	1,87
Deaths	China	Female	Age-standa	Larynx can	Smoking	Rate	2003	0,15
Deaths	China	Male	Age-standa	Larynx can	Smoking	Rate	2004	1,83
Deaths	China	Female	Age-standa	Larynx can	Smoking	Rate	2004	0,15
Deaths	China	Male	Age-standa	Larynx can	Smoking	Rate	2005	1,79
Deaths	China	Female	Age-standa	Larynx can	Smoking	Rate	2005	0,15
Deaths	China	Male	Age-standa	Larynx can	Smoking	Rate	2006	1,68
Deaths	China	Female	Age-standa	Larynx can	Smoking	Rate	2006	0,14
Deaths	China	Male	Age-standa	Larynx can	Smoking	Rate	2007	1,65
Deaths	China	Female	Age-standa	Larynx can	Smoking	Rate	2007	0,13
Deaths	China	Male	Age-standa	Larynx can	Smoking	Rate	2008	1,66
Deaths	China	Female	Age-standa	Larynx can	Smoking	Rate	2008	0,13
Deaths	China	Male	Age-standa	Larynx can	Smoking	Rate	2009	1,66
Deaths	China	Female	Age-standa	Larynx can	Smoking	Rate	2009	0,13
Deaths	China	Male	Age-standa	Larynx can	Smoking	Rate	2010	1,67
Deaths	China	Female	Age-standa	Larynx can	Smoking	Rate	2010	0,12
Deaths	China	Male	Age-standa	Larynx can	Smoking	Rate	2011	1,66
Deaths	China	Female	Age-standa	Larynx can	Smoking	Rate	2011	0,12
Deaths	China	Male	Age-standa	Larynx can	Smoking	Rate	2012	1,66
Deaths	China	Female	Age-standa	Larynx can	Smoking	Rate	2012	0,12
Deaths	China	Male	Age-standa	Larynx can	Smoking	Rate	2013	1,63
Deaths	China	Female	Age-standa	Larynx can	Smoking	Rate	2013	0,11
Deaths	China	Male	Age-standa	Larynx can	Smoking	Rate	2014	1,59
Deaths	China	Female	Age-standa	Larynx can	Smoking	Rate	2014	0,11
Deaths	China	Male	Age-standa	Larynx can	Smoking	Rate	2015	1,58
Deaths	China	Female	Age-standa	Larynx can	Smoking	Rate	2015	0,10
Deaths	China	Male	Age-standa	Larynx can	Smoking	Rate	2016	1,58
Deaths	China	Female	Age-standa	Larynx can	Smoking	Rate	2016	0,10
Deaths	China	Male	Age-standa	Larynx can	Smoking	Rate	2017	1,53
Deaths	China	Female	Age-standa	Larynx can	Smoking	Rate	2017	0,10
Deaths	China	Male	Age-standa	Larynx can	Smoking	Rate	2018	1,49
Deaths	China	Female	Age-standa	Larynx can	Smoking	Rate	2018	0,10
Deaths	China	Male	Age-standa	Larynx can	Smoking	Rate	2019	1,47
Deaths	China	Female	Age-standa	Larynx can	Smoking	Rate	2019	0,10
Deaths	China	Male	Age-standa	Larynx can	Smoking	Rate	2020	1,46
Deaths	China	Female	Age-standa	Larynx can	Smoking	Rate	2020	0,10
Deaths	China	Male	Age-standa	Larynx can	Smoking	Rate	2021	1,44
Deaths	China	Female	Age-standa	Larynx can	Smoking	Rate	2021	0,09
DALYs (D	China	Male	Age-standa	Larynx can	Smoking	Rate	1990	58,87
DALYs (D	China	Female	Age-standa	Larynx can	Smoking	Rate	1990	4,37
DALYs (D	China	Male	Age-standa	Larynx can	Smoking	Rate	1991	58,26
DALYs (D	China	Female	Age-standa	Larynx can	Smoking	Rate	1991	4,38

DALYs (D	China	Male	Age-standa	Larynx can	Smoking	Rate	1992	56,63
DALYs (D	China	Female	Age-standa	Larynx can	Smoking	Rate	1992	4,33
DALYs (D	China	Male	Age-standa	Larynx can	Smoking	Rate	1993	55,67
DALYs (D	China	Female	Age-standa	Larynx can	Smoking	Rate	1993	4,24
DALYs (D	China	Male	Age-standa	Larynx can	Smoking	Rate	1994	54,36
DALYs (D	China	Female	Age-standa	Larynx can	Smoking	Rate	1994	4,16
DALYs (D	China	Male	Age-standa	Larynx can	Smoking	Rate	1995	53,34
DALYs (D	China	Female	Age-standa	Larynx can	Smoking	Rate	1995	4,04
DALYs (D	China	Male	Age-standa	Larynx can	Smoking	Rate	1996	51,85
DALYs (D	China	Female	Age-standa	Larynx can	Smoking	Rate	1996	3,88
DALYs (D	China	Male	Age-standa	Larynx can	Smoking	Rate	1997	50,21
DALYs (D	China	Female	Age-standa	Larynx can	Smoking	Rate	1997	3,72
DALYs (D	China	Male	Age-standa	Larynx can	Smoking	Rate	1998	49,21
DALYs (D	China	Female	Age-standa	Larynx can	Smoking	Rate	1998	3,63
DALYs (D	China	Male	Age-standa	Larynx can	Smoking	Rate	1999	47,94
DALYs (D	China	Female	Age-standa	Larynx can	Smoking	Rate	1999	3,54
DALYs (D	China	Male	Age-standa	Larynx can	Smoking	Rate	2000	47,58
DALYs (D	China	Female	Age-standa	Larynx can	Smoking	Rate	2000	3,49
DALYs (D	China	Male	Age-standa	Larynx can	Smoking	Rate	2001	46,39
DALYs (D	China	Female	Age-standa	Larynx can	Smoking	Rate	2001	3,46
DALYs (D	China	Male	Age-standa	Larynx can	Smoking	Rate	2002	44,92
DALYs (D	China	Female	Age-standa	Larynx can	Smoking	Rate	2002	3,36
DALYs (D	China	Male	Age-standa	Larynx can	Smoking	Rate	2003	43,73
DALYs (D	China	Female	Age-standa	Larynx can	Smoking	Rate	2003	3,27
DALYs (D	China	Male	Age-standa	Larynx can	Smoking	Rate	2004	42,83
DALYs (D	China	Female	Age-standa	Larynx can	Smoking	Rate	2004	3,21
DALYs (D	China	Male	Age-standa	Larynx can	Smoking	Rate	2005	41,68
DALYs (D	China	Female	Age-standa	Larynx can	Smoking	Rate	2005	3,11
DALYs (D	China	Male	Age-standa	Larynx can	Smoking	Rate	2006	39,62
DALYs (D	China	Female	Age-standa	Larynx can	Smoking	Rate	2006	2,94
DALYs (D	China	Male	Age-standa	Larynx can	Smoking	Rate	2007	38,84
DALYs (D	China	Female	Age-standa	Larynx can	Smoking	Rate	2007	2,84
DALYs (D	China	Male	Age-standa	Larynx can	Smoking	Rate	2008	38,96
DALYs (D	China	Female	Age-standa	Larynx can	Smoking	Rate	2008	2,78
DALYs (D	China	Male	Age-standa	Larynx can	Smoking	Rate	2009	38,86
DALYs (D	China	Female	Age-standa	Larynx can	Smoking	Rate	2009	2,71
DALYs (D	China	Male	Age-standa	Larynx can	Smoking	Rate	2010	38,98
DALYs (D	China	Female	Age-standa	Larynx can	Smoking	Rate	2010	2,65
DALYs (D	China	Male	Age-standa	Larynx can	Smoking	Rate	2011	38,87
DALYs (D	China	Female	Age-standa	Larynx can	Smoking	Rate	2011	2,59
DALYs (D	China	Male	Age-standa	Larynx can	Smoking	Rate	2012	38,91
DALYs (D	China	Female	Age-standa	Larynx can	Smoking	Rate	2012	2,48
DALYs (D	China	Male	Age-standa	Larynx can	Smoking	Rate	2013	38,15
DALYs (D	China	Female	Age-standa	Larynx can	Smoking	Rate	2013	2,36
DALYs (D	China	Male	Age-standa	Larynx can	Smoking	Rate	2014	37,17
DALYs (D	China	Female	Age-standa	Larynx can	Smoking	Rate	2014	2,26
DALYs (D	China	Male	Age-standa	Larynx can	Smoking	Rate	2015	36,91
DALYs (D	China	Female	Age-standa	Larynx can	Smoking	Rate	2015	2,20
DALYs (D	China	Male	Age-standa	Larynx can	Smoking	Rate	2016	36,69
DALYs (D	China	Female	Age-standa	Larynx can	Smoking	Rate	2016	2,17

DALYs (D	China	Male	Age-standa	Larynx can	Smoking	Rate	2017	35,81
DALYs (D	China	Female	Age-standa	Larynx can	Smoking	Rate	2017	2,13
DALYs (D	China	Male	Age-standa	Larynx can	Smoking	Rate	2018	34,98
DALYs (D	China	Female	Age-standa	Larynx can	Smoking	Rate	2018	2,09
DALYs (D	China	Male	Age-standa	Larynx can	Smoking	Rate	2019	34,53
DALYs (D	China	Female	Age-standa	Larynx can	Smoking	Rate	2019	2,06
DALYs (D	China	Male	Age-standa	Larynx can	Smoking	Rate	2020	34,23
DALYs (D	China	Female	Age-standa	Larynx can	Smoking	Rate	2020	2,05
DALYs (D	China	Male	Age-standa	Larynx can	Smoking	Rate	2021	33,79
DALYs (D	China	Female	Age-standa	Larynx can	Smoking	Rate	2021	2,01

lower	upper
7292	10936
442	1071
7498	11310
453	1077
7555	11199
463	1096
7746	11338
507	1064
7789	11282
509	1074
7993	11202
522	1088
7892	11335
540	1068
7941	11282
548	1037
8108	11229
541	1057
8188	11165
552	1049
8424	11188
552	1062
8503	11412
558	1096
8540	11055
528	1087
8596	11251
525	1078
8593	11307
551	1109
8742	11246
542	1112
8622	11171
544	1069
8705	11374
544	1070
9089	11608
561	1094
9299	12264
540	1110
9575	12793
564	1136
9950	13392
556	1157
10073	14259
539	1177

10237	14254
520	1145
10277	14629
523	1158
10431	15312
526	1201
10556	15928
530	1256
10370	16400
547	1270
10195	16667
547	1363
10213	17773
574	1428
10274	17793
588	1527
10545	18691
581	1551
202997	308036
10000	25419
208931	318463
10194	25679
209345	314827
10431	25795
214733	316324
11259	24752
215139	314574
11340	25108
221729	310115
11450	24957
217187	314541
12134	24637
218625	310418
12124	23917
222325	309924
11668	24149
222587	307339
12297	24230
228200	302575
12126	24354
230439	312367
12011	24888
230002	302490
11575	24742
230419	303680
11473	24445
230649	307074
12018	25374
234734	303837
12203	25226

233891	301799
12097	24398
233006	306670
12077	24343
243153	314666
12364	24682
246977	327254
12343	24915
252961	341516
12428	25707
260191	352867
12236	25858
260642	375385
12027	26103
266311	374911
11766	25613
264394	383119
11811	25997
265588	396332
11963	26691
269495	412008
11543	27586
261158	425715
12135	27672
255335	433120
12352	29971
258186	462410
12747	31168
257904	458387
12841	33294
260942	474093
12810	33449
1,97	2,90
0,12	0,27
1,97	2,88
0,11	0,26
1,93	2,80
0,12	0,26
1,92	2,75
0,12	0,25
1,88	2,70
0,12	0,25
1,87	2,64
0,12	0,24
1,81	2,58
0,12	0,23
1,78	2,51
0,12	0,22
1,75	2,41
0,12	0,22

1,73	2,36
0,11	0,21
1,74	2,31
0,11	0,21
1,71	2,28
0,11	0,21
1,66	2,13
0,10	0,20
1,64	2,12
0,10	0,19
1,61	2,08
0,10	0,19
1,57	2,00
0,09	0,19
1,48	1,91
0,09	0,17
1,45	1,88
0,09	0,17
1,46	1,85
0,08	0,16
1,45	1,88
0,08	0,16
1,43	1,90
0,08	0,16
1,42	1,92
0,07	0,15
1,41	1,96
0,07	0,15
1,38	1,87
0,06	0,14
1,34	1,87
0,06	0,14
1,31	1,90
0,06	0,14
1,28	1,92
0,06	0,14
1,22	1,88
0,06	0,13
1,16	1,85
0,06	0,14
1,12	1,89
0,06	0,14
1,09	1,83
0,05	0,14
1,08	1,87
0,05	0,14
47,14	70,53
2,41	5,93
46,97	70,66
2,42	5,83

45,96	68,15
2,40	5,70
45,82	66,79
2,50	5,36
44,84	65,23
2,46	5,31
44,97	63,16
2,43	5,16
43,17	62,28
2,50	5,00
42,21	59,98
2,45	4,71
41,76	57,99
2,30	4,66
40,87	56,25
2,35	4,54
40,95	54,21
2,25	4,46
40,14	54,16
2,19	4,43
38,96	50,81
2,03	4,28
38,10	49,73
1,95	4,11
36,87	48,76
1,99	4,13
36,46	46,97
1,95	3,98
35,15	44,91
1,86	3,70
33,91	44,32
1,79	3,58
34,21	43,90
1,78	3,51
33,73	44,39
1,69	3,43
33,28	44,62
1,67	3,39
33,27	44,89
1,58	3,31
32,30	46,21
1,50	3,21
31,91	44,36
1,41	3,03
30,76	44,09
1,35	2,96
30,03	44,30
1,32	2,95
29,52	44,78
1,23	2,93

27,73	44,64
1,25	2,85
26,49	44,04
1,21	2,95
25,91	45,58
1,21	2,97
25,12	44,05
1,18	3,05
24,76	44,48
1,13	2,96

Supplementary Table 2. Joinpoint regression analysis of ASMR (age-standardized mortality rate) and ASDR (age-standardized disability-adjusted life years rate) for laryngeal cancer attributable to smoking in China, 1990-2021.

measure	location	sex	cause	rei	age
Deaths	China	Female	Larynx cancer	Smoking	Age-standardized
Deaths	China	Male	Larynx cancer	Smoking	Age-standardized
DALYs (Disability-Adjusted Life Years)	China	Female	Larynx cancer	Smoking	Age-standardized
DALYs (Disability-Adjusted Life Years)	China	Male	Larynx cancer	Smoking	Age-standardized

measure	location	sex	cause	rei	age
Deaths	China	Female	Larynx cancer	Smoking	Age-standardized
Deaths	China	Female	Larynx cancer	Smoking	Age-standardized
Deaths	China	Female	Larynx cancer	Smoking	Age-standardized
Deaths	China	Female	Larynx cancer	Smoking	Age-standardized
Deaths	China	Female	Larynx cancer	Smoking	Age-standardized
Deaths	China	Female	Larynx cancer	Smoking	Age-standardized
Deaths	China	Male	Larynx cancer	Smoking	Age-standardized
Deaths	China	Male	Larynx cancer	Smoking	Age-standardized
Deaths	China	Male	Larynx cancer	Smoking	Age-standardized
Deaths	China	Male	Larynx cancer	Smoking	Age-standardized
DALYs (Disability-Adjusted Life Years)	China	Female	Larynx cancer	Smoking	Age-standardized
DALYs (Disability-Adjusted Life Years)	China	Female	Larynx cancer	Smoking	Age-standardized
DALYs (Disability-Adjusted Life Years)	China	Female	Larynx cancer	Smoking	Age-standardized
DALYs (Disability-Adjusted Life Years)	China	Female	Larynx cancer	Smoking	Age-standardized
DALYs (Disability-Adjusted Life Years)	China	Female	Larynx cancer	Smoking	Age-standardized
DALYs (Disability-Adjusted Life Years)	China	Female	Larynx cancer	Smoking	Age-standardized
DALYs (Disability-Adjusted Life Years)	China	Male	Larynx cancer	Smoking	Age-standardized
DALYs (Disability-Adjusted Life Years)	China	Male	Larynx cancer	Smoking	Age-standardized
DALYs (Disability-Adjusted Life Years)	China	Male	Larynx cancer	Smoking	Age-standardized
DALYs (Disability-Adjusted Life Years)	China	Male	Larynx cancer	Smoking	Age-standardized

joinpoint	AAPC.Index	Start.Obs	End.Obs	AAPC_95CI	Significant_indicator
5	Full Range	1990	2021	-2.43 (-2.84 to -2.02)	Yes
3	Full Range	1990	2021	-1.69 (-1.87 to -1.52)	Yes
5	Full Range	1990	2021	-2.50 (-2.82 to -2.18)	Yes
3	Full Range	1990	2021	-1.83 (-1.97 to -1.69)	Yes

joinpoint	Segment	Segment.Start	Segment.End	APC_95CI	Significant_indicator
5	0	1990	1994	-1.17 (-2.12 to -0.21)	Yes
5	1	1994	1998	-3.48 (-4.72 to -2.22)	Yes
5	2	1998	2001	-1.18 (-3.66 to 1.36)	No
5	3	2001	2011	-2.95 (-3.19 to -2.71)	Yes
5	4	2011	2014	-4.41 (-7.26 to -1.47)	Yes
5	5	2014	2021	-1.46 (-1.93 to -0.99)	Yes
3	0	1990	2004	-2.03 (-2.12 to -1.94)	Yes
3	1	2004	2007	-3.49 (-4.91 to -2.05)	Yes
3	2	2007	2011	0.46 (-0.31 to 1.24)	No
3	3	2011	2021	-1.53 (-1.72 to -1.33)	Yes
5	0	1990	1994	-1.34 (-2.19 to -0.49)	Yes
5	1	1994	1998	-3.63 (-4.76 to -2.48)	Yes
5	2	1998	2001	-1.36 (-3.60 to 0.94)	No
5	3	2001	2011	-2.98 (-3.19 to -2.76)	Yes
5	4	2011	2015	-3.84 (-5.08 to -2.59)	Yes
5	5	2015	2021	-1.36 (-1.88 to -0.85)	Yes
3	0	1990	2004	-2.31 (-2.38 to -2.23)	Yes
3	1	2004	2007	-3.32 (-4.50 to -2.12)	Yes
3	2	2007	2012	0.03 (-0.40 to 0.46)	No
3	3	2012	2021	-1.61 (-1.81 to -1.41)	Yes

Test.Statistic	P.Value
-11,51	0,00
-18,65	0,00
-15,04	0,00
-25,01	0,00

Test.Statistic	P.Value
-2,60	0,02
-5,84	0,00
-1,00	0,33
-25,77	0,00
-3,18	0,01
-6,54	0,00
-47,63	0,00
-4,97	0,00
1,23	0,23
-16,34	0,00
-3,34	0,00
-6,66	0,00
-1,27	0,23
-29,26	0,00
-6,44	0,00
-5,59	0,00
-63,22	0,00
-5,71	0,00
0,15	0,88
-16,68	0,00

Supplementary Table 3: The impact of age on mortality rates and disability-adjusted life year (DALY) rates of laryngeal cancer attributable to smoking among males and females in China.

measure	location	sex	cause	rei	label	Age	Rate	CILo
Deaths	China	Male	Larynx can	Smoking	30 to 34	32,5	0,07	0,06
Deaths	China	Male	Larynx can	Smoking	35 to 39	37,5	0,21	0,19
Deaths	China	Male	Larynx can	Smoking	40 to 44	42,5	0,54	0,51
Deaths	China	Male	Larynx can	Smoking	45 to 49	47,5	1,06	1,01
Deaths	China	Male	Larynx can	Smoking	50 to 54	52,5	1,95	1,87
Deaths	China	Male	Larynx can	Smoking	55 to 59	57,5	2,92	2,80
Deaths	China	Male	Larynx can	Smoking	60 to 64	62,5	3,96	3,78
Deaths	China	Male	Larynx can	Smoking	65 to 69	67,5	5,09	4,86
Deaths	China	Male	Larynx can	Smoking	70 to 74	72,5	6,59	6,28
Deaths	China	Male	Larynx can	Smoking	75 to 79	77,5	7,45	7,08
Deaths	China	Male	Larynx can	Smoking	80 to 84	82,5	8,56	8,11
Deaths	China	Male	Larynx can	Smoking	85 to 89	87,5	12,94	12,15
Deaths	China	Male	Larynx can	Smoking	90 to 94	92,5	13,47	12,17
Deaths	China	Male	Larynx can	Smoking	95 plus	97,5	7,63	5,47
Deaths	China	Female	Larynx can	Smoking	30 to 34	32,5	0,00	0,00
Deaths	China	Female	Larynx can	Smoking	35 to 39	37,5	0,01	0,00
Deaths	China	Female	Larynx can	Smoking	40 to 44	42,5	0,02	0,01
Deaths	China	Female	Larynx can	Smoking	45 to 49	47,5	0,03	0,02
Deaths	China	Female	Larynx can	Smoking	50 to 54	52,5	0,07	0,05
Deaths	China	Female	Larynx can	Smoking	55 to 59	57,5	0,14	0,12
Deaths	China	Female	Larynx can	Smoking	60 to 64	62,5	0,22	0,18
Deaths	China	Female	Larynx can	Smoking	65 to 69	67,5	0,33	0,26
Deaths	China	Female	Larynx can	Smoking	70 to 74	72,5	0,46	0,37
Deaths	China	Female	Larynx can	Smoking	75 to 79	77,5	0,50	0,40
Deaths	China	Female	Larynx can	Smoking	80 to 84	82,5	0,54	0,43
Deaths	China	Female	Larynx can	Smoking	85 to 89	87,5	0,52	0,41
Deaths	China	Female	Larynx can	Smoking	90 to 94	92,5	0,53	0,39
Deaths	China	Female	Larynx can	Smoking	95 plus	97,5	0,47	0,28
DALYs (D	China	Male	Larynx can	Smoking	30 to 34	32,5	4,30	3,82
DALYs (D	China	Male	Larynx can	Smoking	35 to 39	37,5	11,14	10,37
DALYs (D	China	Male	Larynx can	Smoking	40 to 44	42,5	26,66	25,40
DALYs (D	China	Male	Larynx can	Smoking	45 to 49	47,5	47,19	45,34
DALYs (D	China	Male	Larynx can	Smoking	50 to 54	52,5	77,38	74,67
DALYs (D	China	Male	Larynx can	Smoking	55 to 59	57,5	102,22	98,75
DALYs (D	China	Male	Larynx can	Smoking	60 to 64	62,5	120,22	115,55
DALYs (D	China	Male	Larynx can	Smoking	65 to 69	67,5	130,79	125,49
DALYs (D	China	Male	Larynx can	Smoking	70 to 74	72,5	139,68	133,66
DALYs (D	China	Male	Larynx can	Smoking	75 to 79	77,5	127,15	121,04
DALYs (D	China	Male	Larynx can	Smoking	80 to 84	82,5	114,67	107,96
DALYs (D	China	Male	Larynx can	Smoking	85 to 89	87,5	138,10	127,25
DALYs (D	China	Male	Larynx can	Smoking	90 to 94	92,5	125,30	106,36
DALYs (D	China	Male	Larynx can	Smoking	95 plus	97,5	65,83	36,08
DALYs (D	China	Female	Larynx can	Smoking	30 to 34	32,5	0,17	0,14
DALYs (D	China	Female	Larynx can	Smoking	35 to 39	37,5	0,35	0,30
DALYs (D	China	Female	Larynx can	Smoking	40 to 44	42,5	0,85	0,77
DALYs (D	China	Female	Larynx can	Smoking	45 to 49	47,5	1,24	1,14

DALYs (D	China	Female	Larynx can	Smoking	50 to 54	52,5	2,60	2,44
DALYs (D	China	Female	Larynx can	Smoking	55 to 59	57,5	5,00	4,71
DALYs (D	China	Female	Larynx can	Smoking	60 to 64	62,5	6,73	6,30
DALYs (D	China	Female	Larynx can	Smoking	65 to 69	67,5	8,52	7,97
DALYs (D	China	Female	Larynx can	Smoking	70 to 74	72,5	9,89	9,22
DALYs (D	China	Female	Larynx can	Smoking	75 to 79	77,5	8,61	8,01
DALYs (D	China	Female	Larynx can	Smoking	80 to 84	82,5	7,34	6,78
DALYs (D	China	Female	Larynx can	Smoking	85 to 89	87,5	5,68	5,14
DALYs (D	China	Female	Larynx can	Smoking	90 to 94	92,5	5,06	4,37
DALYs (D	China	Female	Larynx can	Smoking	95 plus	97,5	4,15	3,03

CIHi
0,09
0,23
0,58
1,12
2,04
3,04
4,14
5,33
6,91
7,83
9,05
13,79
14,92
10,64
0,01
0,01
0,02
0,04
0,08
0,17
0,27
0,41
0,57
0,62
0,68
0,67
0,72
0,79
4,83
11,96
27,99
49,11
80,19
105,81
125,09
136,32
145,97
133,58
121,80
149,88
147,62
120,11
0,21
0,40
0,93
1,34

2,78
5,31
7,20
9,12
10,60
9,26
7,95
6,26
5,86
5,68

Supplementary Table 4: The impact of period on mortality rates and disability-adjusted life year (DALY) rates of laryngeal cancer attributable to smoking among males and females in China.

measure	location	sex	cause	rei	label	Period	Rate Ratic	CI Lo
Deaths	China	Male	Larynx can	Smoking	1992 to 19!	1994,5	1,32	1,27
Deaths	China	Male	Larynx can	Smoking	1997 to 20(	1999,5	1,15	1,11
Deaths	China	Male	Larynx can	Smoking	2002 to 20(	2004,5	1,00	1,00
Deaths	China	Male	Larynx can	Smoking	2007 to 20	2009,5	0,91	0,89
Deaths	China	Male	Larynx can	Smoking	2012 to 20	2014,5	0,89	0,86
Deaths	China	Male	Larynx can	Smoking	2017 to 20:	2019,5	0,83	0,79
Deaths	China	Female	Larynx can	Smoking	1992 to 19!	1994,5	1,32	1,18
Deaths	China	Female	Larynx can	Smoking	1997 to 20(	1999,5	1,13	1,02
Deaths	China	Female	Larynx can	Smoking	2002 to 20(	2004,5	1,00	1,00
Deaths	China	Female	Larynx can	Smoking	2007 to 20	2009,5	0,85	0,77
Deaths	China	Female	Larynx can	Smoking	2012 to 20	2014,5	0,73	0,65
Deaths	China	Female	Larynx can	Smoking	2017 to 20:	2019,5	0,67	0,59
DALYs (D	China	Male	Larynx can	Smoking	1992 to 19!	1994,5	1,31	1,25
DALYs (D	China	Male	Larynx can	Smoking	1997 to 20(	1999,5	1,15	1,11
DALYs (D	China	Male	Larynx can	Smoking	2002 to 20(	2004,5	1,00	1,00
DALYs (D	China	Male	Larynx can	Smoking	2007 to 20	2009,5	0,92	0,89
DALYs (D	China	Male	Larynx can	Smoking	2012 to 20	2014,5	0,89	0,86
DALYs (D	China	Male	Larynx can	Smoking	2017 to 20:	2019,5	0,84	0,79
DALYs (D	China	Female	Larynx can	Smoking	1992 to 19!	1994,5	1,34	1,28
DALYs (D	China	Female	Larynx can	Smoking	1997 to 20(	1999,5	1,13	1,09
DALYs (D	China	Female	Larynx can	Smoking	2002 to 20(	2004,5	1,00	1,00
DALYs (D	China	Female	Larynx can	Smoking	2007 to 20	2009,5	0,86	0,82
DALYs (D	China	Female	Larynx can	Smoking	2012 to 20	2014,5	0,73	0,70
DALYs (D	China	Female	Larynx can	Smoking	2017 to 20:	2019,5	0,67	0,64

**CI Hi**
1,36
1,18
1,00
0,94
0,92
0,86
1,47
1,25
1,00
0,94
0,81
0,76
1,37
1,19
1,00
0,95
0,94
0,90
1,40
1,18
1,00
0,89
0,76
0,70

Supplementary Table 5: The impact of birth cohort on mortality rates and disability-adjusted life year (DALY) rates of laryngeal cancer attributable to smoking among males and females in China.

measure	location	sex	cause	rei	label	Cohort	Rate Ratic	CILo
Deaths	China	Male	Larynx can	Smoking	1895 to 18!	1897	1,70	0,39
Deaths	China	Male	Larynx can	Smoking	1900 to 19(	1902	1,61	1,17
Deaths	China	Male	Larynx can	Smoking	1905 to 19(	1907	1,49	1,31
Deaths	China	Male	Larynx can	Smoking	1910 to 19	1912	1,51	1,40
Deaths	China	Male	Larynx can	Smoking	1915 to 19	1917	1,47	1,39
Deaths	China	Male	Larynx can	Smoking	1920 to 19.	1922	1,36	1,30
Deaths	China	Male	Larynx can	Smoking	1925 to 19.	1927	1,27	1,23
Deaths	China	Male	Larynx can	Smoking	1930 to 19.	1932	1,17	1,13
Deaths	China	Male	Larynx can	Smoking	1935 to 19.	1937	1,09	1,06
Deaths	China	Male	Larynx can	Smoking	1940 to 19.	1942	1,00	1,00
Deaths	China	Male	Larynx can	Smoking	1945 to 19.	1947	0,92	0,89
Deaths	China	Male	Larynx can	Smoking	1950 to 19.	1952	0,84	0,81
Deaths	China	Male	Larynx can	Smoking	1955 to 19.	1957	0,76	0,73
Deaths	China	Male	Larynx can	Smoking	1960 to 19(	1962	0,64	0,62
Deaths	China	Male	Larynx can	Smoking	1965 to 19(	1967	0,58	0,55
Deaths	China	Male	Larynx can	Smoking	1970 to 19'	1972	0,47	0,43
Deaths	China	Male	Larynx can	Smoking	1975 to 19'	1977	0,41	0,36
Deaths	China	Male	Larynx can	Smoking	1980 to 19:	1982	0,39	0,31
Deaths	China	Male	Larynx can	Smoking	1985 to 19:	1987	0,37	0,24
Deaths	China	Female	Larynx can	Smoking	1895 to 18!	1897	2,63	0,35
Deaths	China	Female	Larynx can	Smoking	1900 to 19(	1902	2,81	1,50
Deaths	China	Female	Larynx can	Smoking	1905 to 19(	1907	2,51	1,84
Deaths	China	Female	Larynx can	Smoking	1910 to 19	1912	2,23	1,83
Deaths	China	Female	Larynx can	Smoking	1915 to 19	1917	1,80	1,55
Deaths	China	Female	Larynx can	Smoking	1920 to 19.	1922	1,66	1,46
Deaths	China	Female	Larynx can	Smoking	1925 to 19.	1927	1,51	1,34
Deaths	China	Female	Larynx can	Smoking	1930 to 19.	1932	1,34	1,21
Deaths	China	Female	Larynx can	Smoking	1935 to 19.	1937	1,19	1,07
Deaths	China	Female	Larynx can	Smoking	1940 to 19.	1942	1,00	1,00
Deaths	China	Female	Larynx can	Smoking	1945 to 19.	1947	0,82	0,73
Deaths	China	Female	Larynx can	Smoking	1950 to 19.	1952	0,71	0,62
Deaths	China	Female	Larynx can	Smoking	1955 to 19.	1957	0,61	0,51
Deaths	China	Female	Larynx can	Smoking	1960 to 19(	1962	0,50	0,41
Deaths	China	Female	Larynx can	Smoking	1965 to 19(	1967	0,43	0,32
Deaths	China	Female	Larynx can	Smoking	1970 to 19'	1972	0,39	0,25
Deaths	China	Female	Larynx can	Smoking	1975 to 19'	1977	0,36	0,17
Deaths	China	Female	Larynx can	Smoking	1980 to 19:	1982	0,36	0,10
Deaths	China	Female	Larynx can	Smoking	1985 to 19:	1987	0,36	0,04
DALYs (D	China	Male	Larynx can	Smoking	1895 to 18!	1897	1,40	0,08
DALYs (D	China	Male	Larynx can	Smoking	1900 to 19(	1902	1,58	0,90
DALYs (D	China	Male	Larynx can	Smoking	1905 to 19(	1907	1,45	1,17
DALYs (D	China	Male	Larynx can	Smoking	1910 to 19	1912	1,47	1,31
DALYs (D	China	Male	Larynx can	Smoking	1915 to 19	1917	1,43	1,33
DALYs (D	China	Male	Larynx can	Smoking	1920 to 19.	1922	1,33	1,26
DALYs (D	China	Male	Larynx can	Smoking	1925 to 19.	1927	1,25	1,20
DALYs (D	China	Male	Larynx can	Smoking	1930 to 19.	1932	1,16	1,12

DALYs (D	China	Male	Larynx can	Smoking	1935 to 19:	1937	1,09	1,05
DALYs (D	China	Male	Larynx can	Smoking	1940 to 194	1942	1,00	1,00
DALYs (D	China	Male	Larynx can	Smoking	1945 to 194	1947	0,92	0,89
DALYs (D	China	Male	Larynx can	Smoking	1950 to 19:	1952	0,84	0,81
DALYs (D	China	Male	Larynx can	Smoking	1955 to 19:	1957	0,76	0,74
DALYs (D	China	Male	Larynx can	Smoking	1960 to 19(	1962	0,66	0,63
DALYs (D	China	Male	Larynx can	Smoking	1965 to 19(	1967	0,59	0,56
DALYs (D	China	Male	Larynx can	Smoking	1970 to 19'	1972	0,47	0,44
DALYs (D	China	Male	Larynx can	Smoking	1975 to 19'	1977	0,42	0,38
DALYs (D	China	Male	Larynx can	Smoking	1980 to 19:	1982	0,40	0,34
DALYs (D	China	Male	Larynx can	Smoking	1985 to 19:	1987	0,38	0,28
DALYs (D	China	Female	Larynx can	Smoking	1895 to 18!	1897	3,07	0,92
DALYs (D	China	Female	Larynx can	Smoking	1900 to 19(	1902	2,57	1,71
DALYs (D	China	Female	Larynx can	Smoking	1905 to 19(	1907	2,44	2,04
DALYs (D	China	Female	Larynx can	Smoking	1910 to 19	1912	2,19	1,97
DALYs (D	China	Female	Larynx can	Smoking	1915 to 19	1917	1,74	1,62
DALYs (D	China	Female	Larynx can	Smoking	1920 to 19:	1922	1,65	1,56
DALYs (D	China	Female	Larynx can	Smoking	1925 to 19:	1927	1,49	1,42
DALYs (D	China	Female	Larynx can	Smoking	1930 to 19:	1932	1,34	1,29
DALYs (D	China	Female	Larynx can	Smoking	1935 to 19:	1937	1,19	1,14
DALYs (D	China	Female	Larynx can	Smoking	1940 to 194	1942	1,00	1,00
DALYs (D	China	Female	Larynx can	Smoking	1945 to 194	1947	0,83	0,80
DALYs (D	China	Female	Larynx can	Smoking	1950 to 19:	1952	0,72	0,69
DALYs (D	China	Female	Larynx can	Smoking	1955 to 19:	1957	0,62	0,58
DALYs (D	China	Female	Larynx can	Smoking	1960 to 19(	1962	0,52	0,49
DALYs (D	China	Female	Larynx can	Smoking	1965 to 19(	1967	0,45	0,41
DALYs (D	China	Female	Larynx can	Smoking	1970 to 19'	1972	0,40	0,35
DALYs (D	China	Female	Larynx can	Smoking	1975 to 19'	1977	0,34	0,28
DALYs (D	China	Female	Larynx can	Smoking	1980 to 19:	1982	0,27	0,19
DALYs (D	China	Female	Larynx can	Smoking	1985 to 19:	1987	0,25	0,14

CIHi
7,37
2,23
1,70
1,63
1,55
1,41
1,32
1,21
1,13
1,00
0,95
0,86
0,79
0,68
0,62
0,51
0,47
0,50
0,57
19,82
5,28
3,43
2,71
2,10
1,88
1,69
1,50
1,32
1,00
0,93
0,81
0,72
0,63
0,59
0,62
0,74
1,27
3,16
25,58
2,78
1,80
1,65
1,54
1,40
1,31
1,20

1,12
1,00
0,95
0,87
0,79
0,68
0,62
0,50
0,46
0,48
0,51
10,32
3,85
2,93
2,42
1,87
1,74
1,56
1,40
1,24
1,00
0,87
0,75
0,65
0,56
0,49
0,45
0,41
0,38
0,44

Supplementary Table 6: Net drift and local drift values of mortality rates and disability-adjusted life year (DALY) rates for laryngeal cancer attributable to smoking by sex in China.

sex	cause	rei	Net Drift (	CILo	CIHi
Male	Larynx can	Smoking	-1,81	-2,02	-1,60
Female	Larynx can	Smoking	-2,74	-3,34	-2,14
Male	Larynx can	Smoking	-1,73	-2,08	-1,37
Female	Larynx can	Smoking	-2,79	-3,02	-2,57

sex	cause	rei	label	Age	Mean Per	CILo	CIHi
Male	Larynx can	Smoking	30 to 34	32,5	-2,29	-3,56	-1,00
Male	Larynx can	Smoking	35 to 39	37,5	-2,76	-3,46	-2,04
Male	Larynx can	Smoking	40 to 44	42,5	-2,90	-3,31	-2,48
Male	Larynx can	Smoking	45 to 49	47,5	-2,63	-2,89	-2,36
Male	Larynx can	Smoking	50 to 54	52,5	-2,19	-2,38	-2,00
Male	Larynx can	Smoking	55 to 59	57,5	-2,02	-2,18	-1,86
Male	Larynx can	Smoking	60 to 64	62,5	-1,72	-1,87	-1,58
Male	Larynx can	Smoking	65 to 69	67,5	-1,64	-1,78	-1,51
Male	Larynx can	Smoking	70 to 74	72,5	-1,54	-1,68	-1,40
Male	Larynx can	Smoking	75 to 79	77,5	-1,50	-1,68	-1,33
Male	Larynx can	Smoking	80 to 84	82,5	-1,34	-1,58	-1,10
Male	Larynx can	Smoking	85 to 89	87,5	-1,03	-1,43	-0,63
Male	Larynx can	Smoking	90 to 94	92,5	-0,86	-1,80	0,09
Male	Larynx can	Smoking	95 plus	97,5	-0,79	-4,91	3,50
Female	Larynx can	Smoking	30 to 34	32,5	-1,32	-7,85	5,67
Female	Larynx can	Smoking	35 to 39	37,5	-2,07	-5,83	1,83
Female	Larynx can	Smoking	40 to 44	42,5	-2,75	-4,99	-0,45
Female	Larynx can	Smoking	45 to 49	47,5	-3,04	-4,49	-1,57
Female	Larynx can	Smoking	50 to 54	52,5	-3,26	-4,23	-2,29
Female	Larynx can	Smoking	55 to 59	57,5	-3,33	-4,04	-2,62
Female	Larynx can	Smoking	60 to 64	62,5	-3,22	-3,76	-2,67
Female	Larynx can	Smoking	65 to 69	67,5	-3,04	-3,50	-2,58
Female	Larynx can	Smoking	70 to 74	72,5	-2,74	-3,17	-2,29
Female	Larynx can	Smoking	75 to 79	77,5	-2,29	-2,78	-1,80
Female	Larynx can	Smoking	80 to 84	82,5	-2,33	-2,93	-1,72
Female	Larynx can	Smoking	85 to 89	87,5	-2,47	-3,40	-1,53
Female	Larynx can	Smoking	90 to 94	92,5	-2,58	-4,42	-0,71
Female	Larynx can	Smoking	95 plus	97,5	-2,13	-7,74	3,83
Male	Larynx can	Smoking	30 to 34	32,5	-2,27	-3,14	-1,38
Male	Larynx can	Smoking	35 to 39	37,5	-2,70	-3,21	-2,19
Male	Larynx can	Smoking	40 to 44	42,5	-2,85	-3,16	-2,54
Male	Larynx can	Smoking	45 to 49	47,5	-2,57	-2,78	-2,36
Male	Larynx can	Smoking	50 to 54	52,5	-2,13	-2,29	-1,97
Male	Larynx can	Smoking	55 to 59	57,5	-1,94	-2,08	-1,80
Male	Larynx can	Smoking	60 to 64	62,5	-1,67	-1,80	-1,53
Male	Larynx can	Smoking	65 to 69	67,5	-1,57	-1,72	-1,43
Male	Larynx can	Smoking	70 to 74	72,5	-1,46	-1,64	-1,29
Male	Larynx can	Smoking	75 to 79	77,5	-1,40	-1,63	-1,17
Male	Larynx can	Smoking	80 to 84	82,5	-1,25	-1,61	-0,90
Male	Larynx can	Smoking	85 to 89	87,5	-0,95	-1,60	-0,30

Male	Larynx can	Smoking	90 to 94	92,5	-0,82	-2,47	0,85
Male	Larynx can	Smoking	95 plus	97,5	-0,30	-8,31	8,41
Female	Larynx can	Smoking	30 to 34	32,5	-3,05	-4,77	-1,29
Female	Larynx can	Smoking	35 to 39	37,5	-3,12	-4,16	-2,06
Female	Larynx can	Smoking	40 to 44	42,5	-2,95	-3,57	-2,33
Female	Larynx can	Smoking	45 to 49	47,5	-2,98	-3,39	-2,56
Female	Larynx can	Smoking	50 to 54	52,5	-3,12	-3,41	-2,83
Female	Larynx can	Smoking	55 to 59	57,5	-3,21	-3,44	-2,98
Female	Larynx can	Smoking	60 to 64	62,5	-3,14	-3,33	-2,95
Female	Larynx can	Smoking	65 to 69	67,5	-2,97	-3,14	-2,79
Female	Larynx can	Smoking	70 to 74	72,5	-2,68	-2,86	-2,49
Female	Larynx can	Smoking	75 to 79	77,5	-2,18	-2,41	-1,95
Female	Larynx can	Smoking	80 to 84	82,5	-2,22	-2,54	-1,90
Female	Larynx can	Smoking	85 to 89	87,5	-2,36	-2,90	-1,81
Female	Larynx can	Smoking	90 to 94	92,5	-2,32	-3,51	-1,12
Female	Larynx can	Smoking	95 plus	97,5	-2,48	-5,88	1,05

Supplementary Table 7: Projected number of deaths, disability-adjusted life years (DALYs), and corresponding age-standardized rates (ASRs per 100,000) for laryngeal cancer attributable to smoking among males and females in China, 2022-2035.

measure	location	sex	age	cause	rei	metric	year	pred_val
Deaths	China	Male	Age-standa	Larynx can	Smoking	Rate	2022	2,99
Deaths	China	Male	Age-standa	Larynx can	Smoking	Rate	2023	2,94
Deaths	China	Male	Age-standa	Larynx can	Smoking	Rate	2024	2,90
Deaths	China	Male	Age-standa	Larynx can	Smoking	Rate	2025	2,86
Deaths	China	Male	Age-standa	Larynx can	Smoking	Rate	2026	2,81
Deaths	China	Male	Age-standa	Larynx can	Smoking	Rate	2027	2,77
Deaths	China	Male	Age-standa	Larynx can	Smoking	Rate	2028	2,73
Deaths	China	Male	Age-standa	Larynx can	Smoking	Rate	2029	2,69
Deaths	China	Male	Age-standa	Larynx can	Smoking	Rate	2030	2,64
Deaths	China	Male	Age-standa	Larynx can	Smoking	Rate	2031	2,60
Deaths	China	Male	Age-standa	Larynx can	Smoking	Rate	2032	2,56
Deaths	China	Male	Age-standa	Larynx can	Smoking	Rate	2033	2,52
Deaths	China	Male	Age-standa	Larynx can	Smoking	Rate	2034	2,48
Deaths	China	Male	Age-standa	Larynx can	Smoking	Rate	2035	2,44
Deaths	China	Female	Age-standa	Larynx can	Smoking	Rate	2022	0,19
Deaths	China	Female	Age-standa	Larynx can	Smoking	Rate	2023	0,19
Deaths	China	Female	Age-standa	Larynx can	Smoking	Rate	2024	0,19
Deaths	China	Female	Age-standa	Larynx can	Smoking	Rate	2025	0,18
Deaths	China	Female	Age-standa	Larynx can	Smoking	Rate	2026	0,18
Deaths	China	Female	Age-standa	Larynx can	Smoking	Rate	2027	0,18
Deaths	China	Female	Age-standa	Larynx can	Smoking	Rate	2028	0,18
Deaths	China	Female	Age-standa	Larynx can	Smoking	Rate	2029	0,17
Deaths	China	Female	Age-standa	Larynx can	Smoking	Rate	2030	0,17
Deaths	China	Female	Age-standa	Larynx can	Smoking	Rate	2031	0,17
Deaths	China	Female	Age-standa	Larynx can	Smoking	Rate	2032	0,17
Deaths	China	Female	Age-standa	Larynx can	Smoking	Rate	2033	0,16
Deaths	China	Female	Age-standa	Larynx can	Smoking	Rate	2034	0,16
Deaths	China	Female	Age-standa	Larynx can	Smoking	Rate	2035	0,16
DALYs (D	China	Male	Age-standa	Larynx can	Smoking	Rate	2022	68,91
DALYs (D	China	Male	Age-standa	Larynx can	Smoking	Rate	2023	67,79
DALYs (D	China	Male	Age-standa	Larynx can	Smoking	Rate	2024	66,69
DALYs (D	China	Male	Age-standa	Larynx can	Smoking	Rate	2025	65,60
DALYs (D	China	Male	Age-standa	Larynx can	Smoking	Rate	2026	64,51
DALYs (D	China	Male	Age-standa	Larynx can	Smoking	Rate	2027	63,44
DALYs (D	China	Male	Age-standa	Larynx can	Smoking	Rate	2028	62,39
DALYs (D	China	Male	Age-standa	Larynx can	Smoking	Rate	2029	61,37
DALYs (D	China	Male	Age-standa	Larynx can	Smoking	Rate	2030	60,36
DALYs (D	China	Male	Age-standa	Larynx can	Smoking	Rate	2031	59,38
DALYs (D	China	Male	Age-standa	Larynx can	Smoking	Rate	2032	58,42
DALYs (D	China	Male	Age-standa	Larynx can	Smoking	Rate	2033	57,50
DALYs (D	China	Male	Age-standa	Larynx can	Smoking	Rate	2034	56,61
DALYs (D	China	Male	Age-standa	Larynx can	Smoking	Rate	2035	55,75
DALYs (D	China	Female	Age-standa	Larynx can	Smoking	Rate	2022	4,12
DALYs (D	China	Female	Age-standa	Larynx can	Smoking	Rate	2023	4,07
DALYs (D	China	Female	Age-standa	Larynx can	Smoking	Rate	2024	4,01
DALYs (D	China	Female	Age-standa	Larynx can	Smoking	Rate	2025	3,95

DALYs (D	China	Female	Age-standa	Larynx can	Smoking	Rate	2026	3,90
DALYs (D	China	Female	Age-standa	Larynx can	Smoking	Rate	2027	3,85
DALYs (D	China	Female	Age-standa	Larynx can	Smoking	Rate	2028	3,79
DALYs (D	China	Female	Age-standa	Larynx can	Smoking	Rate	2029	3,74
DALYs (D	China	Female	Age-standa	Larynx can	Smoking	Rate	2030	3,69
DALYs (D	China	Female	Age-standa	Larynx can	Smoking	Rate	2031	3,64
DALYs (D	China	Female	Age-standa	Larynx can	Smoking	Rate	2032	3,59
DALYs (D	China	Female	Age-standa	Larynx can	Smoking	Rate	2033	3,54
DALYs (D	China	Female	Age-standa	Larynx can	Smoking	Rate	2034	3,49
DALYs (D	China	Female	Age-standa	Larynx can	Smoking	Rate	2035	3,44
Deaths	China	Male	All ages	Larynx can	Smoking	Number	2022	14623
Deaths	China	Male	All ages	Larynx can	Smoking	Number	2023	14833
Deaths	China	Male	All ages	Larynx can	Smoking	Number	2024	15056
Deaths	China	Male	All ages	Larynx can	Smoking	Number	2025	15281
Deaths	China	Male	All ages	Larynx can	Smoking	Number	2026	15495
Deaths	China	Male	All ages	Larynx can	Smoking	Number	2027	15687
Deaths	China	Male	All ages	Larynx can	Smoking	Number	2028	15868
Deaths	China	Male	All ages	Larynx can	Smoking	Number	2029	16056
Deaths	China	Male	All ages	Larynx can	Smoking	Number	2030	16247
Deaths	China	Male	All ages	Larynx can	Smoking	Number	2031	16434
Deaths	China	Male	All ages	Larynx can	Smoking	Number	2032	16611
Deaths	China	Male	All ages	Larynx can	Smoking	Number	2033	16792
Deaths	China	Male	All ages	Larynx can	Smoking	Number	2034	16993
Deaths	China	Male	All ages	Larynx can	Smoking	Number	2035	17205
Deaths	China	Female	All ages	Larynx can	Smoking	Number	2022	1054
Deaths	China	Female	All ages	Larynx can	Smoking	Number	2023	1076
Deaths	China	Female	All ages	Larynx can	Smoking	Number	2024	1100
Deaths	China	Female	All ages	Larynx can	Smoking	Number	2025	1125
Deaths	China	Female	All ages	Larynx can	Smoking	Number	2026	1149
Deaths	China	Female	All ages	Larynx can	Smoking	Number	2027	1172
Deaths	China	Female	All ages	Larynx can	Smoking	Number	2028	1195
Deaths	China	Female	All ages	Larynx can	Smoking	Number	2029	1220
Deaths	China	Female	All ages	Larynx can	Smoking	Number	2030	1244
Deaths	China	Female	All ages	Larynx can	Smoking	Number	2031	1269
Deaths	China	Female	All ages	Larynx can	Smoking	Number	2032	1293
Deaths	China	Female	All ages	Larynx can	Smoking	Number	2033	1318
Deaths	China	Female	All ages	Larynx can	Smoking	Number	2034	1345
Deaths	China	Female	All ages	Larynx can	Smoking	Number	2035	1373
DALYs (D	China	Male	All ages	Larynx can	Smoking	Number	2022	359492
DALYs (D	China	Male	All ages	Larynx can	Smoking	Number	2023	362176
DALYs (D	China	Male	All ages	Larynx can	Smoking	Number	2024	364767
DALYs (D	China	Male	All ages	Larynx can	Smoking	Number	2025	367173
DALYs (D	China	Male	All ages	Larynx can	Smoking	Number	2026	369263
DALYs (D	China	Male	All ages	Larynx can	Smoking	Number	2027	370865
DALYs (D	China	Male	All ages	Larynx can	Smoking	Number	2028	372100
DALYs (D	China	Male	All ages	Larynx can	Smoking	Number	2029	373174
DALYs (D	China	Male	All ages	Larynx can	Smoking	Number	2030	374089
DALYs (D	China	Male	All ages	Larynx can	Smoking	Number	2031	374830
DALYs (D	China	Male	All ages	Larynx can	Smoking	Number	2032	375361
DALYs (D	China	Male	All ages	Larynx can	Smoking	Number	2033	375916

DALYs (D	China	Male	All ages	Larynx can	Smoking	Number	2034	376633
DALYs (D	China	Male	All ages	Larynx can	Smoking	Number	2035	377375
DALYs (D	China	Female	All ages	Larynx can	Smoking	Number	2022	22967
DALYs (D	China	Female	All ages	Larynx can	Smoking	Number	2023	23425
DALYs (D	China	Female	All ages	Larynx can	Smoking	Number	2024	23897
DALYs (D	China	Female	All ages	Larynx can	Smoking	Number	2025	24368
DALYs (D	China	Female	All ages	Larynx can	Smoking	Number	2026	24824
DALYs (D	China	Female	All ages	Larynx can	Smoking	Number	2027	25253
DALYs (D	China	Female	All ages	Larynx can	Smoking	Number	2028	25664
DALYs (D	China	Female	All ages	Larynx can	Smoking	Number	2029	26072
DALYs (D	China	Female	All ages	Larynx can	Smoking	Number	2030	26472
DALYs (D	China	Female	All ages	Larynx can	Smoking	Number	2031	26863
DALYs (D	China	Female	All ages	Larynx can	Smoking	Number	2032	27243
DALYs (D	China	Female	All ages	Larynx can	Smoking	Number	2033	27629
DALYs (D	China	Female	All ages	Larynx can	Smoking	Number	2034	28032
DALYs (D	China	Female	All ages	Larynx can	Smoking	Number	2035	28435

pred_low	pred_up
2,88	3,09
2,79	3,10
2,68	3,12
2,57	3,14
2,45	3,18
2,33	3,21
2,20	3,25
2,08	3,29
1,95	3,34
1,81	3,39
1,68	3,44
1,55	3,49
1,41	3,54
1,28	3,59
0,18	0,21
0,18	0,21
0,17	0,21
0,16	0,21
0,15	0,21
0,15	0,21
0,14	0,21
0,13	0,22
0,12	0,22
0,11	0,22
0,11	0,22
0,10	0,23
0,09	0,23
0,08	0,23
66,09	71,72
64,13	71,45
61,94	71,44
59,58	71,62
57,09	71,93
54,53	72,35
51,92	72,87
49,27	73,46
46,60	74,12
43,91	74,84
41,21	75,62
38,53	76,46
35,85	77,36
33,19	78,31
3,92	4,33
3,79	4,34
3,65	4,37
3,50	4,41

3,34	4,46
3,17	4,53
2,99	4,59
2,81	4,67
2,63	4,75
2,45	4,83
2,26	4,92
2,08	5,01
1,89	5,10
1,70	5,19
13461	15785
13510	16157
13485	16627
13381	17180
13193	17797
12918	18456
12574	19162
12179	19932
11731	20762
11225	21644
10655	22566
10032	23552
9363	24623
8638	25772
827	1282
839	1315
848	1354
853	1398
853	1447
846	1499
835	1557
819	1622
799	1692
773	1767
741	1848
704	1935
663	2031
616	2134
327045	391938
326990	397361
325383	404151
322094	412251
317079	421446
310332	431398
302147	442053
292829	453519
282460	465719
271077	478584
258694	492029
245532	506300

231690	521576
217058	537691
20460	25475
20656	26194
20735	27059
20676	28060
20473	29175
20124	30383
19644	31684
19053	33090
18350	34594
17534	36192
16604	37882
15569	39690
14429	41634
13170	43700

© 2025 Gu X. et al.
